# Referral patterns in the CKD.QLD Registry: A call for revisiting the definition of late referral

**DOI:** 10.1371/journal.pone.0338001

**Published:** 2026-08-26

**Authors:** Clyson Mutatiri, Angela Ratsch, Matthew McGrail, Sree Krishna Venuthurupalli, Srinivas Kondalsamy-Chennakesavan

**Affiliations:** 1 Renal Medicine, Bundaberg Base Hospital, Wide Bay Hospital and Health Service, Bundaberg Central, Queensland, Australia; 2 Rural Clinical School, Faculty of Health, Medicine and Behavioural Sciences, The University of Queensland, Bundaberg, Queensland, Australia; 3 Research Services, Wide Bay Hospital and Health Service, Hervey Bay, Queensland, Australia; 4 Rural Clinical School, Faculty of Health, Medicine and Behavioural Sciences, The University of Queensland, Hervey Bay, Queensland, Australia; 5 Rural Clinical School, Faculty of Health, Medicine and Behavioural Sciences, The University of Queensland, Rockhampton, Queensland, Australia; 6 Rural Clinical School, Faculty of Health, Medicine and Behavioural Sciences, The University of Queensland, Ipswich, Queensland, Australia; 7 Kidney Service, Department of Medicine, West Moreton Hospital and Health Service, Ipswich, Queensland, Australia; 8 Rural Clinical School, Faculty of Health, Medicine and Behavioural Sciences, The University of Queensland, Toowoomba, Queensland, Australia; 9 School of Health and Medical Sciences, University of Southern Queensland, Toowoomba, Queensland, Australia; Chulalongkorn University Faculty of Medicine, THAILAND

## Abstract

**Background:**

Timely nephrology referral is considered important in chronic kidney disease (CKD), however, definitions of “late referral” vary, and may not accurately reflect patient risk or predict outcomes.

**Study aim:**

To evaluate referral patterns among Chronic Kidney Disease Queensland Registry (CKD.QLD) participants, focusing on the timing and appropriateness of referrals and their association with clinical outcomes.

**Methods:**

We conducted a retrospective cohort study of adults (≥18 years) in the CKD.QLD Registry from seven public nephrology clinics (May 2011-June 2018). Participants were followed from referral to kidney replacement therapy (KRT), death, or study end. Late referral was defined as initiation of KRT within 12 months of referral among those who progressed to KRT. Comorbidity burden was assessed using an unweighted count. A primary Cox proportional hazards model was performed in KRT starters. To address selection bias, a multivariable Fine-Gray competing risks model with left truncation was applied to the full cohort, incorporating pre-KRT mortality.

**Results:**

Among 3,775 participants, 775 (20.5%) developed end-stage kidney disease and 513 of these (66.2%) initiated KRT. Overall, 722 (19.1%) died, including 598 before KRT. Late referral occurred in 60 (11.7%) KRT patients. In a fully adjusted Cox proportional hazards model restricted to the 513 participants who progressed to KRT, late referral was not associated with post-KRT mortality (hazard ratio [HR] 0.91; 95% CI 0.52–1.59; p = 0.74). On the other hand, in a fully adjusted Fine-Gray model, longer pre-KRT care showed a statistically significant but clinically small increase in post-KRT mortality (sub-distribution hazard ratio [SHR]1.004 per additional month; 95% CI 1.001–1.006; p = 0.004). This minimal effect was supported by near-overlapping cumulative incidence curves across 1-, 3-, 6-, and 12-month referral thresholds. In contrast, baseline clinical factors were strongly associated with post-KRT mortality: higher comorbidity score (SHR 2.113; 95% CI 1.792–2.493; p < 0.001), pre-existing cardiovascular disease (SHR 1.910; 95% CI 1.232–2.961; p = 0.004), and Indigenous status (SHR 1.931; 95% CI 1.193–3.127; p = 0.007).

**Conclusion:**

After accounting for pre-dialysis deaths, longer pre-KRT care had minimal impact on post-dialysis survival. Outcomes were instead driven mainly by baseline comorbidity and cardiovascular disease rather than referral timing. These findings support a shift from primarily using time-based referral criteria toward risk-stratified approaches in CKD management.

## Introduction

Chronic kidney disease (CKD) is a globally recognised contributor to increasing mortality and morbidity, with an estimated worldwide prevalence of 13.4% [1]. The burden of CKD is disproportionately borne by low-income countries and socio-economically disadvantaged populations [2]. With increasing life expectancy, coupled with increasing prevalence of key risk factors such as diabetes mellitus (DM) and hypertension, CKD has emerged as a critical public health concern and drain on resources for health systems globally [3]. Early identification and intervention are crucial for slowing the progression of CKD and improving patient outcomes.

Early-stage CKD is predominately managed within primary care settings [4]. However, a subset of these patients will ultimately require referral to nephrology clinics for specialist input, particularly as their disease advances or if they develop complications [5]. Traditionally, the referral of individuals with CKD from primary care to specialist nephrology services has been guided by established protocols, such as those outlined by international bodies including Kidney Disease: Improving Global Outcomes (KDIGO) [6], Kidney Health Australia (KHA) [7], and other globally recognised guidelines. The timing of referral (which in this manuscript refers to the date of first encounter with the nephrology clinic) has historically been considered a critical factor influencing outcomes, with the evidence demonstrating that earlier referral allows for better preparation for kidney replacement therapy (KRT), optimised management of comorbidities, and improved survival. Late referral generally refers to patients who had commenced KRT such as dialysis or kidney transplantation within a short period following their initial nephrology clinic visit [8]. Different time frames have been used to define late referral, ranging from starting KRT within 1, 3, 4, 6 months of referral to starting within 12 months of referral. While there is no universally agreed-upon timeframe, this operational time-based definition is widely utilised in clinical studies [9].

Multiple studies have reported that late referral is associated with worse clinical outcomes, including higher mortality rates, increased rates of hospitalisation, and poorer quality of life [10,11]. These adverse outcomes have been attributed to insufficient pre-dialysis education, suboptimal management of anaemia, mineral bone disorder, and delayed preparation for vascular access or transplantation [12]. However, the landscape of CKD management has evolved considerably in recent years. Advances in the understanding of CKD pathophysiology, the introduction of a working system based on estimated glomerular filtration rate (eGFR) and albuminuria, and the recognition of albuminuria as an important marker for early detection and risk stratification have all contributed to improved care [13,14]. The emergence of renoprotective pharmacological therapies, such as renin-angiotensin-aldosterone system (RAAS) inhibitors, sodium-glucose co-transporter-2 (SGLT2) inhibitors, and statins, has enabled primary care clinicians to modify the risk of CKD progression, cardiovascular disease (CVD), and mortality at an earlier stage [15]. As a result, risk factor modification and the application of guideline-based therapy are increasingly being implemented in primary care settings before referral to nephrology [16]. The focus has shifted from simply managing eGFR thresholds to a more holistic approach that includes blood pressure control, optimising glycaemic management in diabetes, and the use of agents that offer renoprotection [17,18].

Given this evolution in CKD care, the question arises: does the timing of referral to nephrology clinics still matter as a determinant of outcomes, particularly death following the initiation of KRT? To address this, we conducted a retrospective analysis of participants enrolled in the Chronic Kidney Disease Queensland Registry (CKD.QLD) to evaluate whether late referral continues to impact outcomes in patients who progressed to KRT. Additionally, the study seeks to critically evaluate and potentially refine the current operational definition of late referral from primary care to nephrology services.

## Methods

### Data source and methods of data collection

The Chronic Kidney Disease in Queensland (CKD.QLD) was established as a collaborative of most public sector kidney care practices in Queensland in May 2011. The CKD.QLD data collection methods have been described by Venuthurapalli *et al*, briefly however, the main objective of the registry was to profile participants with CKD, promoting CKD surveillance, practice improvement and research within the kidney care practice network in the public health system in QLD [19]. Data linkage framework was used to centralise data captured by multiple mechanisms to an individual participant via a unique identifier. This enabled further characterisation of participants with CKD, providing a comprehensive view of the health services utilisation and costs and outcomes of people with CKD.

Adult participants aged 18 years and above with the diagnosis of CKD presenting to public ambulatory nephrology clinics in the QLD health (QH) system were consented to have their demographic and clinical data collected. More than 7,600 participants consented to be part of the Registry between June 2011 and June 2018. Excluded were patients on KRT and those with acute kidney injury (AKI) unless they subsequently developed and met the diagnostic criteria for CKD. Participants were followed until the start of KRT, death or 30 June 2018. For the current study, we excluded participants whose first date of visit to the nephrology clinic couldn’t be established.

The demographic variables recorded included age, gender, ethnicity and geographical location, identified by postcode, as well as laboratory and clinical variables and outcome measures. Variables of interest which were not already in the database were manually collected from integrated electronic Medical Record (ieMR), the Hospital Based Corporate Information System (HBCIS), other QLD health platforms, and from private pathology laboratories as appropriate, following granting of a Public Health Act (PHA) approval. Where only urine protein-to-creatinine ratio (uPCR) and dipstick protein were available, the equations developed by Sumida et al were employed to calculate the predicted urine albumin-to-creatinine ratio (UACR) [20]. The eGFR was calculated from serum creatinine using the Chronic Kidney Disease Epidemiology Collaboration (CKD-EPI) equation. Data extraction was undertaken between 18 January 2021 and 15 June 2025 in compliance with ethics and PHA approvals.

### Study design and population

This retrospective cohort study utilised data from participants aged ≥18 years enrolled in the CKD.QLD Registry between 1 May 2011 and 30 June 2018 from seven public healthcare nephrology clinics. A description of participants and basic study outcomes were previously detailed in a published protocol [21].

### Outcomes and variables

Primary outcomes included progression to end-stage kidney disease (ESKD), commencement of KRT, and mortality before and after commencement of KRT. Demographic and clinical variables were investigated for their potential role as predictors or covariates of these outcomes. Referral timing to nephrology services was assessed and defined by the conventional method as the interval between the first encounter with the nephrology service and commencement of KRT. Late referral was defined as initiation of KRT within 12 months of the initial specialist nephrology referral among patients who progressed to KRT. This threshold reflects the time typically required for clinical optimisation, patient education, and establishment of permanent vascular access [22]. To assess the robustness of this definition, a sensitivity analysis was performed using a multivariable left-truncated Fine-Gray model, with referral duration modelled continuously and compared at 1, 3, 6, and 12 months of pre-KRT care (Figure S3 in S1 File).

The proportion of participants meeting the KHA eGFR referral threshold (<30 mL/min) at referral was summarised descriptively. This threshold was selected over other criteria (e.g., eGFR decline or microscopic haematuria) as eGFR-based cut-offs have traditionally guided key CKD management decisions, including specialist referral, access planning, and transplant evaluation, and because urine microscopy data were unavailable for most participants [23]. The Kidney Failure Risk Equation (KFRE) was applied to participants with CKD stages G3-G5 at referral with available UACR data to estimate 5-year risk of progression to KRT. KFRE risk distributions were summarised descriptively to explore alignment across risk thresholds. Model discrimination and calibration were not assessed.

Socio-Economic Indexes for Areas (SEIFA) Relative Socio-economic Disadvantage (IRSD) scores as produced by the Australian Bureau of Statistics were used to measure socioeconomic status of the enrolled participants by their residential postcode [24]. The IRSD deciles were regrouped into quintiles (lowest, low, middle, high, and highest) with the highest quintile representing the least disadvantaged postal areas and the lowest quintile representing the most disadvantaged postal areas [25]. Participants were further categorised into 3 levels of socio-economic status by combining the two lowest quintiles into the low socio-economic status group, the two highest quintiles into the high socio-economic status group, and the middle quintile into the middle socio-economic status group. Participants with a recorded BMI were divided into obese (BMI ≥ 30) and non-obese (BMI < 30). A modified and unweighted version Charlson Comorbidity Score (also referred to as a simple count of comorbid conditions) was used to predict mortality risk. The weighted Charlson Comorbidity Index (CCI) could not be used because the registry did not contain sufficiently detailed information on the individual conditions required to calculate the score. While the original CCI assigns weights (1–6) to different conditions, research indicates that simply counting the number of conditions often provides similar predictive power for long-term mortality [26]. The CCI scores were classified into four groups based on the distribution in the study population: low = 2 (scored by ESKD per se), moderate = 3–4, high = 5–6, and very high > 6 [27].

### Data analysis

Descriptive statistics and basic inferential statistics were employed to summarise demographic and clinical characteristics, exploring basic data patterns and describing referral patterns. Categorical variables were presented as proportions (numbers and percentages) and continuous variables as means with standard deviations.

For the primary analysis, Cox proportional hazards regression was used. This analysis was restricted to participants who initiated KRT (n = 513), with time zero defined as the date of KRT initiation; deaths before KRT were excluded. Covariates for the full pre-specified model were chosen based on prior clinical knowledge of mortality risk in ESKD, and pre-specified confounder set was retained regardless of univariate significance [28]. The proportional hazards assumption was assessed using Schoenfeld residuals and no significant violations were observed for the overall model (p = 0.79) or for individual covariates. Results are presented as hazard ratios (HRs) with 95% confidence intervals (CIs). Kaplan-Meier curves and log-rank tests were used to compare unadjusted survival between early and late referral groups.

To address potential biases from restricting analysis to patients who reached KRT, a complementary competing risks analysis was conducted in the full cohort (n = 3,775). A Fine-Gray subdistribution hazards model with left-truncation (delayed entry) was applied. Time zero was defined as the date of initial nephrology referral, allowing deaths before KRT (n = 598) to be included as competing events. Patients entered the risk set at the time of KRT initiation and followed up until the end of the study period (post-KRT deaths = 124; censored = 389). Statistically, truncation reduces instability from diminishing risk sets at longer durations and limits potential violations of proportional hazards assumptions. It also improves visual clarity by preserving early event dynamics and enhancing interpretability of cumulative incidence functions. The multivariable model included duration of pre-KRT care, age, sex, Indigenous status, social status score, comorbidity score, and histories of DM, hypertension, CVD, and coronary artery disease. The proportional hazards assumption for the subdistribution model was assessed using time-varying covariate interactions with log time. Adjusted cumulative incidence functions (CIF) were generated using stcurve to compare post-KRT mortality across referral durations of 1, 3, 6, and 12 months (Figure S3 in S1 File). All analyses were performed using Stata (version 18/19), with statistical significance defined as p < 0.05.

### Ethical considerations

The CKD.QLD registry and the hospital record(s) were examined by the research team retrospective to the participants’ details being entered into CKD.QLD and their hospital admissions. Both CKD.QLD data and hospital records contained identifiable information essential for data linkage. At enrolment, participants provided informed consent for CKD.QLD, which included permission to access and link relevant clinical information, such as medical history, pathology reports, and prior hospital admissions. Due to the large number of participants, obtaining individual consent for this study was not feasible. Instead, researchers obtained approval from the CKD.QLD Data Custodian to use existing consent arrangements under HREC/15/QRBW/294 (ERM: 13429) (AM05). Ethics approval was granted by the Royal Brisbane and Women’s Hospital Human Research Ethics Committee in January 2021 (LNR/2020/QRBW/69707, 14/01/2021) to access hospital records using unit record numbers provided by CKD.QLD for matching, with the intention to publish the findings. The requirement for individual consent was waived by the Ethics Committee, as all participants had previously consented to the use of their data for research upon registry enrolment. Additionally, a Public Health Act approval (QCOS/029817/ RD006802) was granted in August 2023 for the release of missing patient data held by the contributing Queensland Health Hospital and Health Services.

## Results

### Study sample characteristics

Figure S1 in S1 File outlines the participant selection process. A total of 3,775 participants were included in the final analysis with mean age of 62.5 ± 15.1 years. Of these, 2,069 (54.8%) were male and 327 (8.7%) identified as Indigenous Australians (Table 1).

**Table 1:** Baseline characteristics at the time of referral and outcomes.

Characteristics	Total sample	Stage 1	Stage 2	Stage 3A	Stage 3B	Stage 4	Stage 5
Participants, n (%)	3775	7.9%	11.0%	18.6%	31.0%	26.2%	5.3%
**Demographics**							
Age, mean	62.5	48.2	57.6	62.5	65.3	65.1	64.3
Gender, male n (%)	2,069 (54.8)	46.1	56.0	57.8	52.6	56.5	58.8
Indigenous, n (%)	327 (8.7)	32	49	52	86	89	19
**SES Status**							
Low status n (%)	1806 (47.8)	48.2	52.2	49.9	47.4	45.4	45.7
Middle status n (%)	1006 (26.7)	25.9	24.5	25.6	26.8	28.1	28.1
High status n (%)	963 (25.5)	25.9	23.3	24.5	25.8	26.5	26.1
BMI, n (mean)	2500 (31.7)	31.6	32.2	31.5	31.9	31.6	30.9
**Primary Renal disease**							
DN, n (%)	1,066 (28.2)	27.6	30.8	29.9	26.9	27.8	28.6
GN, n (%)	473 (12.5)	12.5	12.0	11.4	13.2	12.5	14.1
GRD, n (%)	183 (4.9)	3.7	12.0	4.3	5.4	5.2	4.5
RV, n (%)	985 (26.1)	23.9	28.4	28.6	27.0	23.1	25.6
UNC, n (%)	403 (10.7)	10.8	8.4	9.8	11.4	12.2	6.5
Other, n (%)	665 (17.6)	21.6	15.9	16.1	16.3	19.3	20.6
**Comorbidities**							
DM, n (%)	1,694 (44.9)	32.0	42.8	46.0	47.1	46.3	44.2
HTN, n (%)	2,926 (77.5)	77.7	76.7	74.8	77.4	80.7	78.9
CVD, n (%)	1,538 (40.7)	21.2	32.2	41.1	43.4	47.4	37.7
CAD, n (%)	886 (23.5)	16.8	19.2	23.3	25.0	26.1	23.5
Obesity, n (%)	1,366 (54.6)	51.2	56.6	53.5	56.9	54.0	49.1
**Comorbidity Score**							
Low, n (%)	1223 (32.4)	34.2	32.4	37.2	29.7	32.2	29.5
Moderate, n (%)	1093 (29.0)	26.5	31.1	28.3	28.7	26.9	29.0
High n (%)	765 (20.3)	20.1	17.3	17.5	20.9	22.5	21.5
Very high, n (%)	694 (18.4)	19.1	19.2	17.1	20.7	15.7	20.0
**Laboratory Parameters**							
eGFR, mean	42.4	90	71.5	51.0	36.7	22.9	11.4
UACR, mean	56.1	44.2	68.5	48.6	44.5	66.5	86.8
**Outcomes**							
ESKD, n (%)	775 (20.5)	5.7	11.8	12.4	16.4	34.4	54.8
KRT, n (%)	513 (13.6)	3.7	8.4	8.8	10.6	23.4	34.7
Total death, n (%)	722 (19.1)	5.7	10.6	15.1	18.2	27.1	36.7
Death without KRT, n (%)	598 (15.8)	5.7	10.1	12.7	15.7	21.1	29.2

SD: standard deviation; BMI: body mass index; DN: diabetic nephropathy; GN: glomerulonephritis; GRD: genetic renal disease; RV: renovascular disease; UNC: unknown cause; DM: diabetes mellitus; HTN: hypertension; CVD: cardiovascular disease; CAD: coronary artery disease; eGFR: estimated glomerular filtration rate; UACR: urine albumin-to-creatinine ratio; ESKD: end-stage kidney disease; KRT: kidney replacement therapy.

Socioeconomic status, as measured by the IRSD, revealed that a significant portion of the participants (1,806, 47.8%) lived in the most disadvantaged postal areas (low socioeconomic status) (Table 1). This group represented the largest proportion of participants across all stages of CKD (Table 1).

At the time of referral, nearly half of the participants, 1,872 (49.6%) were in stage G3 CKD (G3A and G3B), whereas 713 (18.9%) were in stages 1 or 2 (eGFR of ≥60 ml/min/1.73m^2^). CKD stages G4 and 5 accounted for 1,190 (31.5%) participants, predominately G4 (Table 1). UACR data were available for 2,605 (69%) participants, with 920 (35.3%) exhibiting a UACR of ≥ 30 mg/mmol (Table S1 in S1 File).

There were 17 types of comorbidities recorded, with the number of comorbidities ranging from 0 to 12 per participant. A large proportion of participants (67.6%) fell into the moderate to very high comorbidity categories, with these groups distributed evenly across all CKD stages (Table 1). Of these 1,737 (68.1%) had an eGFR of ≥30 ml/min at referral (Table S2 in S1 File). Hypertension was the most common comorbidity, present in 77.5% of participants, followed by DM and cardiovascular disease (CVD), 44.9% and 40.7% respectively (Table 1).

### Referral thresholds and outcomes

Around one third of participants (1,194, 31.5%) fulfilled the KHA eGFR threshold (<30ml/min) for specialist referral (Table S2 in S1 File). Among the 2,581 (68.4%) who did not fulfill the eGFR threshold, 216 (8.4%) progressed to KRT while 331 (12.8%) died without KRT (Table S2 in S1 File). Similarly, though 1,685 (64.7%) participants did not fulfil the UACR threshold of ≥30 mg/mmol for referral, of these 191 (11.3%) progressed to KRT while 221 (13.1%) died without KRT (Table S1 in S1 File).

Of the 2,605 participants with both eGFR and UACR data, 371 (14.2%) met both referral criteria, and 150 (40.4%) subsequently required KRT (Table S3 in S1 File). Conversely, 1,203 (46.2%) did not meet either threshold for referral, yet 79 (6.6%) of these went on to require KRT at some point during the period of follow up (Table S3 in S1 File).

### Mortality and progression to ESKD

During the follow up period, 722 (19.1%) participants died, with the majority (598, 82.8%) dying prior to commencing KRT, while 775 (21%) participants progressed to ESKD, of whom 513 (66.2%) received KRT (Table 1). More than half of all deaths in the KRT group (63, 50.8%) occurred among participants from the low socioeconomic status group (Table S4 in S1 File). In the univariate Cox model, late referral was associated with a 27% lower risk of mortality (HR 0.73; 95% CI 0.43–1.26; p = 0.27). In the multivariable model adjusting for all variables except comorbidity score, the estimated mortality risk for late referral decreased to 14% (HR 0.86; 95% CI 0.49–1.49; p = 0.58) (Table S5 in S1 File). When the comorbidity score was added, the late referral estimate further decreased to 9% (HR 0.91; 95% CI 0.52–1.59; p = 0.74). The absence of an observed adverse effect of late referral on KRT mortality in our study is likely explained by confounding from comorbidity burden (HR 1.14; 95% CI 0.92–1.41; p = 0.27) (Table 2). The Kaplan–Meier curve indicated longer post-KRT survival in the late-referred group (Fig. S2 in S1 File); however, the log-rank test showed no statistically significant difference in survival between referral groups (p = 0.26). (Table S6 in S1 File). Advancing age was associated with increased mortality rate, with each 5-year increment corresponding to a 24% increase in the hazard of death (HR = 1.24; 95% CI 1.14–1.35; p < 0.01). Additionally, the presence of cardiovascular disease was associated with a significant increase in mortality rate (HR = 1.72; 95% CI 1.18–2.52; p = 0.01) (Table 2).

**Table 2:** Univariate and multivariable Cox regression analyses (N = 513).

Predictor	Hazard ratio		[95% CI]		p-value	
	MV	UV	MV	UV	MV	UV
Age at KRT start	1.24	1.22	1.14–1.35	1.13–1.32	<0.01	<0.01
Gender (male)	1.15	1.17	0.78–1.67	0.81–1.69	0.48	0.39
Indigenous	1.47	1.26	0.85–2.54	0.79–2.02	0.17	0.33
Diabetes Mellitus	1.22	1.69	0.83–1.78	1.19–2.41	0.31	<0.01
Cardiovascular Disease	1.72	1.91	1.18–2.52	1.32–2.76	0.01	<0.01
Hypertension	1.28	1.47	0.73–2.22	0.86–2.52	0.39	0.16
Coronary Artery Disease	1.30	1.19	0.88–1.92	0.82–1.73	0.18	0.36
Primary Renal Disease	1.00	1.01	0.91–1.11	0.91–1.11	0.94	0.87
Social Status	1.09	0.97	0.75–1.58	0.80–1.19	0.66	0.79
Late referral	0.91	0.73	0.52–1.59	0.43–1.26	0.74	0.27
Comorbidity Score (mCCI)						
Moderate	4.60	4.34	0.62–34.45	0.58–32.18	0.14	0.15
High	5.56	5.49	0.76–40.67	0.76–39.88	0.09	0.09
Very high	5.26	5.13	0.72–38.35	0.71–37.03	0.10	0.11
Aggregate Comorbidity Score	1.14	1.15	0.92–1.41	0.94–1.42	0.23	0.18

KRT: kidney replacement therapy; mCCI: modified Charlson Comorbidity Score; MV: multivariable; UV: Univariate.

### Complementary competing-risks and sensitivity analysis

In the left-truncated competing risks analysis of the full cohort (n = 3,775), deaths occurring before KRT (n = 598; 15.8%) were included as competing events for post-KRT mortality (n = 124). After accounting for pre-KRT mortality as a competing risk in the fully adjusted, unrestricted Fine-Gray model, longer duration of pre-KRT care (time under care) showed a statistically significant but clinically minimal effect on post-KRT outcomes (SHR 1.0038 per additional month; 95% CI 1.0012–1.0064; p = 0.004). This corresponds to a 0.38% relative change in hazard per month, or approximately 4.7% over one year of care. However, mortality risk was primarily associated with baseline clinical factors. Each one-unit increase in comorbidity score was associated with more than a twofold increase in mortality risk (SHR 2.113; 95% CI 1.792–2.493; p < 0.001). Pre-existing cardiovascular disease (SHR 1.910; 95% CI 1.232–2.961; p = 0.004) and Indigenous status (SHR 1.931; 95% CI 1.193–3.127; p = 0.007) were also independently associated with increased risk. Age, sex, DM, hypertension, coronary artery disease, and social status were not independently associated (Table 3). To assess the impact of different referral thresholds, adjusted cumulative incidence functions were generated for 1, 3, 6, and 12 months of pre-KRT care (Figure S3 in S1 File). The resulting curves were closely aligned, indicating minimal differences in risk across these thresholds after accounting for competing risks and comorbidities.

**Table 3:** Final multivariable fine-gray competing-risks regression for post-KRT mortality (N = 3,775).

Covariate	SHR	95% CI	p-value
**time_under_care (per month)**	**1.004**	**1.001–1.006**	**0.004**
Age at Referral (per year)	1.016	0.949–1.088	0.654
Gender (Male)	1.389	0.970–1.989	0.072
**Indigenous Status**	**1.931**	**1.193–3.127**	**0.007**
Social Status Score	1.070	0.864–1.326	0.536
**Overall Comorbidity Score**	**2.113**	**1.792–2.493**	**< 0.001**
**Cardiovascular Disease**	**1.910**	**1.232–2.961**	**0.004**
Diabetes Mellitus (DM)	0.951	0.655–1.379	0.789
Hypertension	1.409	0.809–2.453	0.225
Coronary Artery Disease (CAD)	1.119	0.760–1.648	0.568

Abbreviations: CI, confidence interval; KRT, kidney replacement therapy; SHR, subdistribution hazard ratio.

Note: Time Zero is anchored at the initial nephrology referral date to capture pre-KRT mortality (n = 598) as a competing risk event. Follow-up time was left-truncated at KRT initiation and censored at death or the end of the study. Total failures observed post-KRT = 124.

### Referral timing and comorbidity

Of the 513 participants who commenced KRT, 60 (11.7%) were classified as late referrals. Among these, 32 (53.3%) were from low socioeconomic group, compared to 20 (33.3%) from high socioeconomic group (Table 4). Most participants who started KRT (397, 77.4%) (Table 4) and most who died (473, 65.5%) (Table S7 in S1 File) were in the moderate to very high comorbidity groups. Of those who died after starting KRT, (123, 99.2%) were from the moderate to the very high comorbidity groups, most of whom (108, 87.8%) had been referred early (Table S8 in S1 File). Only 15 (12.1%) of those who died post-KRT were late referrals, with 3 (20%) of these dying in the first 12 months of starting KRT (Table 4). The proportion of participants who progressed to KRT was similar across the moderate (15.2%), high (15.6%), and very high (16.1%) comorbidity groups, and all were higher than the proportion in the low comorbidity group (9.5%) (Fig. S4 in S1 File). Among those who progressed to KRT, the mortality rate increased progressively with higher comorbidity scores (Fig. S5 in S1 File). These findings suggest that comorbidity burden may be a stronger predictor of death than late referral alone.

**Table 4:** Characteristics of participants who progressed to KRT.

	Total N = 513	Early Referral N = 453	Late Referral N = 60	P-Value
Parameters at time of referral				
Age (mean, SD)	56.1 ± 13.9	56.1 ± 13.8	56.2 ± 15.2	0.06
Gender (male) n (%)	317 (61.8)	276 (60.9)	41 (68.3)	0.27
Indigenous, n (%)	76 (14.8)	69 (15.2)	7 (11.7)	0.47
Social status				0.94
Low status, n (%)	261 (50.9)	229 (50.6)	32 (53.3)	
Middle status, n (%)	107 (20.9)	99 (21.9)	8 (13.3)	
High status, n (%)	145 (28.3)	125 (27.6)	20 (33.3)	
BMI Category				0.06
< 30, n (%)	127 (45.2)	105 (43.0)	22 (59.5)	
≥ 30, n (%)	154 (54.8)	139 (57.0)	15 (40.5)	
Primary Renal disease				0.09
DN, n (%)	196 (38.2)	169 (37.3)	27 (45)	
GN, n (%)	83 (16.2)	71 (15.7)	12 (20)	
GRD, n (%)	51 (9.9)	46 (10.2)	5 (8.3)	
RV, n (%)	90 (17.5)	81 (17.9)	9 (15.0)	
UNC, n (%)	24 (4.68)	23 (5.1)	1 (1.7)	
Other, n (%)	69 (13.5)	63 (13.9)	6 (10)	
Comorbidities				
DM, n (%)	166 (32.4)	144 (31.8)	22 (36.7)	0.05
HTN, n (%)	435 (84.8)	390 (86.1)	45 (75)	0.02
CVD, n (%)	278 (54.2)	247 (54.5)	31 (51.7)	0.68
CAD, n (%)	151 (29.4)	138 (30.5)	13 (21.7)	0.16
Comorbidity Score				0.58
Low, n (%)	116 (22.6)	102 (22.5)	14 (23.3)	
Moderate, n (%)	166 (32.4)	152 (33.6)	14 (23.3)	
High, n (%)	119 (23.2)	102 (22.5)	17 (28.3)	
Very High, n (%)	112 (21.8)	97 (21.4)	15 (25.0)	
Death after KRT, n (%)	124 (24.2)	109 (24.1)	15 (25.0)	0.87
Within 12 months, n (%)	26 (21.0)	23 (21.1)	3 (20.0)	
≥ 12 months < 24 months, n (%)	30 (24.2)	28 (25.7)	2 (13.3)	
≥ 24 months, n (%)	68 (54.8)	58 (53.2)	10 (66.7)	
Laboratory Parameters				
eGFR (mean, SD)	31.0 ± 17.6	31.9 ± 17.6	24.3 ± 16.2	<0.01
UACR (mean, SD)	102.8 ± 145.4	102.0 ± 145.7	109 ± 144.0	
Risk Classification				
eGFR < 30, n (%)	297 (57.9)	253 (55.9)	44 (73.3)	
eGFR ≥ 30, n (%)	216 (42.1)	200 (44.2)	16 (26.7)	
UACR < 30, n (%)	191 (41.5)	171 (41.9)	20 (38.5)	
UACR ≥ 30, n (%)	269 (58.5)	237 (58.1)	32 (61.5)	
5-Year KFRE < 5, n (%)	73 (15.9)	66 (16.2)	7 (13.5)	
5-Year KFRE ≥ 5, n (%)	387 (84.1)	342 (83.8)	45 (86.5)	

SD: standard deviation; BMI: body mass index; DN: diabetic nephropathy; GN: glomerulonephritis; GRD: genetic renal disease; RV: renovascular disease; UNC: unknown cause; DM: diabetes mellitus; HTN: hypertension; CVD: cardiovascular disease; CAD: coronary artery disease; eGFR: estimated glomerular filtration rate; UACR: urine albumin-to-creatinine ratio; ESKD: end-stage kidney disease; KFRE: kidney failure risk equation.

### CKD stage and risk of progression

Participants in CKD stages 1–3 were found to be at greater risk of death before KRT than progress to KRT (55.4% vs 43.5%), whereas those in stages 4 & 5 were more likely to initiate KRT than die before KRT (56.5% vs 44.6%) (Table 1). Overall, mortality rates increased with advancing CKD stages (Table 1).

### KFRE application and risk of KRT progression

Among the 1,743 participants who did not meet the KHA eGFR referral threshold and had available UACR data, descriptive cross-tabulation using 5-year KFRE risk thresholds classified 708 (40.6%) and 509 (29.2%) patients at the 3% and 5% risk levels, respectively. Within these groups, 144 (20.3%) and 126 (24.8%) progressed to KRT, while 91 (12.9%) and 65 (12.8%) died without receiving KRT (Table S9 in S1 File). Among participants who met the KHA eGFR threshold, KFRE classification identified a similar proportion of patients who progressed to KRT at both the 3% (99.6%) and 5% (96.6%) thresholds. The proportions progressing to KRT or dying were also similar across these thresholds (KRT: 31.0% and 31.9%; death: 15.6% and 14.9%; Table S10 in S1 File).

These descriptive findings suggest that the eGFR threshold and KFRE risk categories showed similar overall patterns in cohort distribution for KRT progression and pre-KRT mortality. However, KFRE additionally identified a group of higher-risk patients who were not captured by the eGFR threshold alone.

## Discussion

In our primary restricted analysis, we observed that participants who were in the care of nephrology clinics for more than 12 months before commencing KRT showed a non-significant trend toward lower post-KRT survival rates than those referred within 12 months (late referrals) (HR: 0.91; p = 0.74). While this trend initially appeared to contrast with historical studies that have universally reported poorer survival rates in patients referred late, these older cohorts frequently predate the widespread adoption of structured contemporary CKD management in primary care and hence may reflect outdated practice patterns [29–31]. However, our primary analysis suffered from some methodological limitations common to retrospective kidney care literature where the design introduces important bias. By restricting follow-up strictly to patients surviving to initiate KRT, the analysis is affected by survivor bias and potential collider bias, as both referral timing and baseline health status influence survival to dialysis.

To address these limitations, we performed a complementary analysis using a multivariable Fine-Gray model with left truncation across the full registry cohort (N = 3,775). Follow-up was anchored at the time of initial nephrology referral, and pre-KRT deaths (n = 598, 15.8%) were treated as a competing risk. In this framework, longer pre-KRT care was associated with a statistically significant but clinically small increase in post-KRT mortality risk corresponding to a 0.38% relative increase in hazard per month, or approximately 4.7% over one year of care. Sensitivity analyses across multiple time thresholds (1, 3, 6, and 12 months) showed near-identical cumulative incidence curves, indicating that varying time-based definitions of referral may not meaningfully change risk estimates once confounding and competing risks are addressed. These results support the interpretation that time under care may not be an independent predictor but instead reflects underlying disease severity and progression.

This finding is not consistent with current clinical understanding that longer nephrology care improves post-dialysis outcomes and suggests that duration of care alone may not adequately explain survival after initiation of KRT.

Previous research has highlighted that even patients who have been under nephrology care for longer periods may still undergo urgent start dialysis [32–34]. Moreover, nephrology care does not always ensure optimal dialysis preparation. For example, Hamadah and Gharaibeh [35] found that only 30.8% of patients under nephrology care for more than 12 months started haemodialysis with an arteriovenous fistula (AVF). Similarly, Mendelssohn, Curtis [36] demonstrated that many patients who were followed by a nephrologist experienced suboptimal dialysis commencement, defined as using a temporary central dialysis catheter access for haemodialysis or peritoneal dialysis immediately after placement of a PD catheter. A follow-up study by the same group replicated the results of their study by demonstrating a 56.4% rate of suboptimal starts in early referred patients [37].

Importantly, we observed that the effect of care duration was modest compared with the impact of baseline patient factors. Comorbidity burden was strongly associated with mortality (SHR 2.113 per unit increase, p < 0.001), and cardiovascular disease independently increased risk (SHR 1.910, p = 0.004). There was a high burden of comorbidities in all participants, most of whom had an eGFR of ≥30 ml/min at the time of referral. This trend was maintained in participants who progressed to KRT; a higher burden of comorbidities was observed in those who were referred early (commenced KRT after 12 months of referral) and in those who died after commencing KRT. These results suggest that participants who had more comorbid conditions were more readily referred early to nephrology practice rather than stable patients, whose main reason of referral would have been uncomplicated CKD. Preexisting comorbidities have been reported to predict higher mortality rates in patients starting dialysis [38]. In a prospective cohort study of patients with CKD stage G5 and delayed initiation of haemodialysis, patients with a high and very high CCI were found to have an increased risk for all-cause mortality, with mortality risk increasing progressively as the CCI score increased [39].These findings suggest that underlying health status and structural factors depicted by high comorbidity rate may be the main determinant of long-term outcomes, superseding the impact of late referral to nephrology services. Among participants with available data, 46.3% did not meet the KHA eGFR or UACR referral thresholds. This distribution suggests that many of these patients were referred for other clinical reasons or may not have met standard criteria and could potentially have been managed in primary care settings. In a similar retrospective cohort study of CKD patients over 14 years, Ghimire, Ye [40] observed guideline discordant rate of 59% and concluded that many of the referred patients being seen by nephrologists might have had mild CKD that could have been safely managed in primary care. Bikbov, Purcell [41] reported that adherence to the KDIGO guidelines would result in a supply-demand mismatch and concluded that implementing the guidelines may not be feasible. Kiel, Weckmann [42] came to a similar conclusion, reporting that implementing KDIGO criteria would lead to more than double increase in referral rate compared to actual referral. Current referral criteria are not designed to stratify patients according to future risk. In our cohort, descriptive cross-tabulation showed that a substantial proportion of participants who did not meet the eGFR referral threshold were classified within higher risk categories when assessed using 5-year KFRE thresholds (3% and 5%). Within this group, the KFRE categories showed a closer correspondence with the proportions of participants who progressed to KRT compared with those who died.

The timing of participant follow-up in our study should be considered in the context of evolving kidney care practices. Improvements in CKD management within primary care may partly explain the less pronounced associations observed in our findings. Enhanced education of primary care providers, improved disease definition and staging, and the integration of guideline-based therapies have collectively contributed to better patient outcomes. The widespread use of new pharmacological agents, particularly those with proven renoprotective and cardioprotective effects, has further lessened the reliance on specialist referral for optimal care [43].

Our findings should not be interpreted as evidence that delayed referral is safe or equivalent to early referral. Importantly, this study was observational and was not designed to evaluate the effectiveness of delayed referral, selective referral, or primary-care-only management strategies. Consequently, causal conclusions regarding the optimal timing of referral cannot be drawn. Furthermore, important aspects of nephrology care that may influence outcomes, including vascular access planning, dialysis modality education, transplantation assessment, and management of CKD complications, were not captured within the current analysis.

The study does, however, raise questions regarding the utility of retrospective time-based definitions of late referral. Historically, referral timeliness has been defined using arbitrary intervals before KRT initiation, ranging from one month to one year. Our sensitivity analyses demonstrated minimal differences across these thresholds after accounting for competing risks and baseline comorbidity. This observation suggests that fixed temporal definitions may not adequately reflect the heterogeneity of risk among patients with CKD.

In parallel, descriptive analyses demonstrated that a substantial proportion of patients who did not meet traditional eGFR-based referral thresholds nevertheless progressed to KRT, while others who met referral criteria experienced competing outcomes such as death before KRT. These findings highlight the limitations of relying solely on kidney function thresholds to characterise future risk. Risk-prediction tools such as the KFRE may provide additional information regarding progression risk and therefore warrant further evaluation in referral frameworks.

Nevertheless, the present findings should be regarded as hypothesis-generating. While risk-stratification approaches may offer a promising means of identifying patients at greatest risk of CKD progression, our study did not directly compare risk-based referral strategies against existing referral practices. Accordingly, our results should not be interpreted as supporting changes to current referral pathways or the withholding of specialist care from lower-risk patients.

A key strength of this study is the inclusion of participant characteristics at the time of nephrology referral, in contrast to prior studies that focus on data at KRT initiation. The use of a multivariable competing risks approach with a left-truncated Fine-Gray model (n = 3,775), with time zero defined as referral, enabled inclusion of deaths prior to KRT (n = 598) as competing events while maintaining focus on post-KRT outcomes by allowing entry into the risk set at KRT initiation. Inclusion of both measured and estimated UACR values supported sufficient sample size for descriptive analysis, with baseline 5-year KFRE scores used as an exploratory risk proxy to compare risk categories with observed progression without formal evaluation of predictive performance.

This study has several limitations. The retrospective design introduces potential residual confounding and reverse causality. The post-KRT analysis included relatively few late-referral patients, limiting statistical precision. Restriction to participants who progressed to KRT may introduce selection (collider) bias, as both referral timing and baseline health status influence progression. Missing data, particularly for UACR, were handled using listwise deletion, which may bias results if missingness was non-random. Comorbidity burden was assessed using an unweighted count, which does not capture differences in severity. Key clinical data, including referral indications, dialysis modality, vascular access, and cause of death, were unavailable. The cohort was limited to public nephrology clinics in Queensland, potentially limiting generalisability.

### The case for a new definition of late referral: A risk-based approach

We propose that CKD referral timeliness should be prospectively redefined using risk stratification at the time of initial nephrology consultation. Current retrospective definitions, which classify referral timing according to variable intervals ranging from 1 to 12 months before KRT initiation, lack consensus, contribute to heterogeneity across studies, and offer limited value for clinical decision-making. In contrast, our findings suggest that baseline patient characteristics, particularly comorbidity burden and cardiovascular disease, are more strongly associated with post-KRT mortality than the conventional time-based measures of referral timing used in many previous studies.

Future prospective studies are needed to determine whether incorporating validated risk-prediction tools, such as the KFRE, at the time of referral can improve referral prioritisation and resource allocation while maintaining patient safety and equitable access to specialist care. Such an approach could enable clinicians to identify high-risk patients who may benefit from early vascular access planning and modality preparation, while allowing lower-risk patients to be managed with a greater emphasis on disease stabilisation, comorbidity management, and cardiovascular risk reduction.

Until such evidence is available, risk-based approaches should be considered a promising area for further investigation rather than a basis for changing current referral practices. Although these findings require confirmation in additional studies, they support consideration of a shift towards risk-based definitions of referral timeliness and CKD management.

## Conclusions

After accounting for pre-dialysis mortality to minimise survival bias, longer pre-KRT nephrology care was associated with a statistically significant but clinically small increase in post-KRT mortality risk. In contrast, baseline comorbidity burden, cardiovascular disease, and Indigenous status were the strongest predictors of post-KRT mortality.

These findings suggest that retrospective, time-based definitions of referral timeliness have limited prognostic value within this cohort. However, the observational nature of this study precludes conclusions regarding the safety or effectiveness of delayed referral strategies or alternative referral pathways.

## Supporting information

S1 File Supplementary tables, figures, and supporting analyses. Contains Tables S1-S10 and Figures S1-S5, including outcome distributions by eGFR and UACR categories, comorbidity analyses, survival analyses, competing-risk analyses, and KFRE subgroup comparisons.
(DOCX)

S2 File Underlying data for study analyses (CSV). Dataset used to generate the analyses and results reported in this study.
(CSV)
