# Supplementary material for: Referral patterns in the CKD.QLD Registry: A call for revisiting the definition of late referral
Source: PLoS One. 2026 Aug 26;21(8):e0338001. doi: 10.1371/journal.pone.0338001 (PMC13514023; doi:10.1371/journal.pone.0338001)
Supplement: Supplementary file 1 [file pone.0338001.s001.docx]

**S1 File. Supporting Information**

**Table S1** Distribution of KRT and Death without KRT by UACR categories

| KHA threshold | N = 2605 | KRT | Death without KRT |
| --- | --- | --- | --- |
| UACR <30 | 1685 (64.7%) | 191 (11.3%) | 221 (13.1%) |
| UACR ≥30 | 920 (35.3%) | 216 (8.4%) | 331 (12.8%) |

**Table S1 Legend:** Distribution of KRT initiation and death without KRT by UACR categories defined according to KHA thresholds. Data are presented as number (percentage) within each UACR category. The total study population comprised 2,605 participants, grouped as UACR <30 mg/g and ≥30 mg/g. Outcomes include incidence of KRT and death prior to KRT initiation.

Abbreviations: KRT, kidney replacement therapy; UACR, urine albumin-to-creatinine ratio; KHA, Kidney Health Australia.

**Table S2** Distribution of outcomes and comorbidity scores by eGFR categories

| KHA threshold | All participants  N (%) | KRT  n (%) | Death without KRT  n (%) | Low  comorbidity  n (%) | Moderate  comorbidity  n (%) | High comorbidity  n (%) | Very High comorbidity  n (%) |
| --- | --- | --- | --- | --- | --- | --- | --- |
| eGFR <30 | 1194 (31.6%) | 297 (24.9%) | 267 (22.4%) | 379 (31.7%) | 352 (29.5%) | 267 (22.4) | 196 (16.4) |
| eGFR ≥30 | 2581 (68.4%) | 216 (8.4%) | 331 (12.8%) | 844 (69.0%) | 741 (28.7%) | 498 (19.3) | 498 (19.3) |
| Total | 3775 | 513 | 598 | 1223 | 1093 | 765 | 694 |

**Table S2 Legend:** Distribution of clinical outcomes and comorbidity burden by eGFR categories defined according to KHA thresholds. Data are presented as number (percentage) unless otherwise specified. Participants are stratified into eGFR <30 and ≥30 mL/min/1.73 m². Outcomes include initiation of KRT and death without KRT. Comorbidity burden is categorized as low, moderate, high, and very high. Totals are provided for the full study cohort (N = 3,775).

Abbreviations: eGFR, estimated glomerular filtration rate; KRT, kidney replacement therapy; KHA, Kidney Health Australia.

**Table S3** Distribution of KRT by eGFR and UACR categories

|  | UACR category (number of KRT) | | |
| --- | --- | --- | --- |
| eGFR | <30 mg-mmol | ≥30 mg-mol | Total |
| <30 ml-min | 482 (112) | 371 (150) | 853 (262) |
| >=30 ml-min | 1203 (79) | 549 (119) | 1752 (198) |
| Total | 1685 (191) | 920 (269) | 2605 (460) |

**Table S3 Legend:** Distribution of KRT events by combined eGFR and UACR categories. Data are presented as total number of participants within each category, with the number of KRT events shown in parentheses. eGFR categories are stratified as <30 and ≥30 mL/min, and UACR categories as <30 and ≥30 mg/mmol. Totals are provided across all categories for the full cohort (N = 2,605).

Abbreviations: KRT, kidney replacement therapy; eGFR, estimated glomerular filtration rate; UACR, urine albumin-to-creatinine ratio.

**Table S4** Distribution of death by timing of referral and socioeconomic status

| Timing of referral | Death in Low SES | Death in Middle SES | Death in High SES |
| --- | --- | --- | --- |
| Early referral | 55 | 25 | 29 |
| Late referral | 8 | 2 | 5 |
| Total Death | 63 | 27 | 34 |

**Table S4 Legend:** Distribution of deaths by timing of referral and SES. Data are presented as the number of deaths within each SES category (low, middle, and high), stratified by early and late referral groups. Totals are provided for deaths across SES categories.

Abbreviation: SES, socio-economic status

**Table S5.** **Multivariable Cox regression analysis of factors associated with post-KRT mortality, excluding comorbidity score.**

| **Predictor** | **Hazard Ratio** | **95% CI** | **p-value** |
| --- | --- | --- | --- |
| **Late referral (<12 months)** | 0.86 | 0.49-1.49 | 0.58 |
| **Age at KRT initiation** | 1.24 | 1.14-1.35 | <0.01 |
| **Male sex** | 1.16 | 0.79 - 1.70 | 0.45 |
| **Indigenous status** | 1.44 | 0.84 - 2.47 | 0.19 |
| **Diabetes mellitus** | 1.25 | 0.86 - 1.82 | 0.25 |
| **Cardiovascular disease** | 1.74 | 1.19 - 2.54 | <0.01 |
| **Hypertension** | 1.23 | 0.71 - 2.13 | 0.47 |
| **Coronary artery disease** | 1.28 | 0.87 - 1.88 | 0.21 |
| **Peripheral vascular disease** | 1.01 | 0.91 - 1.11 | 0.90 |
| **Social status** | 0.100 | 0.81 - 1.22 | 0.96 |

**Table S5 Legend:** Hazard ratios estimate the association between baseline demographic and clinical characteristics and post-KRT mortality among participants who progressed to kidney replacement therapy. The model excludes comorbidity score and includes referral timing, age at KRT initiation, sex, Indigenous status, diabetes, cardiovascular disease, hypertension, coronary artery disease, peripheral vascular disease, and social status. HR >1 indicates increased mortality risk, while HR <1 indicates reduced mortality risk.

Abbreviations: CI, confidence interval; KRT, kidney replacement therapy.

**Table S6** Log-rank test for equality of survivor functions by referral status

| Timing of referral | Observed events | Expected events |
| --- | --- | --- |
| ≥12 months \| | 109 | 105 |
| <12 months | 15 | 19 |
| Total | 124 | 124 |

*chi2(1) =	1.25; Pr > chi2 = 0.26*

**Table S6 Legend:** Log-rank test comparing equality of survivor functions by timing of referral. Participants are stratified into referral ≥12 months and <12 months. The table presents observed and expected numbers of events for each group, with totals shown for the full cohort. The log-rank test statistic (χ² = 1.25, p = 0.26) indicates no statistically significant difference in survival between referral groups.

**Figure S3:** Predicted cumulative incidence of post-KRT death stratified by duration of pre‑KRT nephrology care

**Figure S3 Legend**
This figure displays the predicted cumulative incidence of death following initiation of KRT over the follow up period after referral to nephrology care. Estimates were derived from an unrestricted multivariable Fine-Gray competing risks model that accounts for pre‑KRT attrition. The x‑axis represents time from nephrology care entry (from referral to end of study), and the y‑axis represents the absolute probability of post‑KRT death.

Separate curves correspond to different durations of pre‑KRT care prior to KRT initiation: 1 month (solid blue line), 3 months (dashed red line), 6 months (dash‑dot green line), 12 months (dotted orange line), and 24 months (purple line). Across all groups, the cumulative incidence of post‑KRT death increases gradually over time, reaching approximately 7-8% at 10 years. The trajectories are closely aligned, with only modest separation between groups at later time points, indicating minimal differences in predicted post‑KRT mortality across varying durations of pre‑KRT nephrology care.

Abbreviation: KRT, kidney replacement therapy.

**Table S7** Distribution of total death by comorbidity score

| Death | Low | Moderate | High | Very high |
| --- | --- | --- | --- | --- |
| No | 974 | 884 | 624 | 571 |
| Yes | 249 | 209 | 141 | 123 |

**Table S7 Legend:** Distribution of mortality by comorbidity burden. Data are presented as the number of participants with and without death (Yes/No), stratified by comorbidity categories (low, moderate, high, and very high). Totals within each comorbidity category reflect the distribution of deaths and survivors in the study cohort.

**Table S8** Distribution of KRT death by comorbidity score and referral status

| Comorbidity score | KRT Death | | Referral Status | |
| --- | --- | --- | --- | --- |
|  | No | Yes | ≥ 12 months | < 12 months |
| Low | 15 | 1 | 1 | 0 |
| Moderate | 78 | 22 | 21 | 1 |
| High | 124 | 42 | 37 | 5 |
| Very high | 172 | 59 | 50 | 9 |
| Total | 389 | 124 | 109 | 15 |

**Table S8 Legend:** Distribution of kidney replacement therapy (KRT)-related deaths by comorbidity score and timing of referral. Data are presented as the number of participants with and without KRT death (Yes/No), stratified by comorbidity categories (low, moderate, high, and very high). Referral status is categorized as ≥12 months and <12 months prior to the event. Totals are provided for all categories.

Abbreviation: KRT, kidney replacement therapy

**S4 Figure.** Distribution of kidney replacement therapy (KRT) progression by comorbidity score

**Figure S4 Legend:** This bar chart illustrates the distribution of progression to KRT according to comorbidity score categories (low, moderate, high, and very high). The x-axis represents comorbidity score categories, and the y-axis shows the percentage of participants within each category.

For each comorbidity level, participants are categorized as either not progressing to KRT (blue bars) or progressing to KRT (orange bars). The majority of participants in all categories did not progress to KRT; however, the proportion of KRT progression increases progressively with higher comorbidity burden. Specifically, KRT progression rose from 9.5% in the low comorbidity group to 15.2% in the moderate group, 15.6% in the high group, and 16.1% in the very high group. Correspondingly, the proportion of participants not progressing to KRT decreased from 90.5% to 83.9% across these categories.

These findings suggest a modest positive association between increasing comorbidity burden and the likelihood of progression to KRT.

Abbreviation: KRT, kidney replacement therapy

**S5 Figure.** Distribution of post–kidney replacement therapy (KRT) survival and death by comorbidity score

**Figure S5 Legend:** This bar chart depicts the distribution of survival and death outcomes following KRT across comorbidity score categories (low, moderate, high, and very high). The x-axis represents comorbidity score categories, and the y-axis shows the number of participants in each outcome group.

For each comorbidity category, participants are grouped into those who survived (blue bars) and those who died (orange bars). The absolute number of deaths increases progressively with higher comorbidity burden: from 1 in the low group to 22 in the moderate group, 42 in the high group, and 59 in the very high group. Similarly, the number of survivors also increases across categories (15, 78, 124, and 172, respectively), reflecting a larger overall population in higher comorbidity groups.

Abbreviations: KRT, kidney replacement therapy.

**Table S9** Comparison of eGFR threshold and KFRE Subgroup of participants with eGFR of ≥30ml/min

| N = 1743 | | KFRE <3% | KFRE ≥3% | KFRE <5% | KFRE ≥5% |
| --- | --- | --- | --- | --- | --- |
| Baseline | | 1035 (59.4%) | 708 (40.6%) | 1234 (70.8%) | 509 (29.2%) |
| KRT | Yes | 54 (5.2%) | 144 (20.3%) | 72 (5.8%) | 126 (24.8%) |
|  | No | 981 (94.8%) | 564 (79.7%) | 1162 (94.2%) | 383 (75.2%) |
| Death | Yes | 117 (11.3%) | 91 (12.9%) | 143 (11.6%) | 65 (12.8%) |
|  | No | 918 (88.7%) | 578 (81.6%) | 1077 (88.4%) | 444 (87.2%) |

**Table S9 Legend:** Comparison of eGFR threshold and KFRE risk categories among participants with baseline eGFR ≥30 mL/min (N = 1,743). Data are presented as number (percentage) within KFRE risk groups defined by 3% and 5% thresholds. Outcomes include kidney replacement therapy (KRT) and death, stratified by KFRE risk categories.

Abbreviations: eGFR, estimated glomerular filtration rate; KFRE, kidney failure risk equation; KRT, kidney replacement therapy

**Table S10** Comparison of eGFR threshold and KFRE Subgroup of participants with eGFR of <30ml/min

| N = 847 | | KFRE <3% | KFRE ≥3% | KFRE <5% | KFRE ≥5% |
| --- | --- | --- | --- | --- | --- |
| Baseline | | 16 (3%) | 844 (99.6%) | 29 (3.4%) | 818 (96.6%) |
| KRT | Yes | 0 (0%) | 262 (31.0%) | 1 (3.4%) | 261 (31.9%) |
|  | No | 16 (100%) | 582 (69.0%) | 28 (96.6%) | 557 (68.1%) |
| Death | Yes | 0 (0%) | 132 (15.6) | 10 (34.5%) | 122 (14.9%) |
|  | No | 3 (100%) | 712 (84.4%) | 19(65.5%) | 696 (85.1%) |

**Table S10 Legend:** Comparison of eGFR threshold and KFRE risk categories among participants with baseline eGFR <30 mL/min (N = 847). Data are presented as number (percentage) within KFRE risk groups defined by 3% and 5% thresholds. Outcomes include KRT and death, stratified by KFRE risk categories.

Abbreviations: eGFR, estimated glomerular filtration rate; KFRE, kidney failure risk equation; KRT, kidney replacement therapy
