# Supplementary material for: Referral patterns in the CKD.QLD Registry: A call for revisiting the definition of late referral
Source: PLoS One. 2026 Aug 26;21(8):e0338001. doi: 10.1371/journal.pone.0338001 (PMC13514023; doi:10.1371/journal.pone.0338001)
Supplement: Supplementary file 2 [file pone.0338001.s003.docx]

Summary and general comments

Mutatiri et al. present a study evaluating referral patterns among CKD.QLD Registry participants, focusing on the timing and appropriateness of referrals and their association with clinical outcomes. However, while the topic is interesting and locally relevant, the study design, data analysis, and interpretation of the results raise several methodological concerns that must be addressed before the paper can be considered for publication.

Suggestions for major revisions

Review Comments to the Author

Issues that need to be addressed include the following:

Major Comments

1. Overall significance and interpretation of the main finding

- The finding that “late referral” (defined as ≤12 months before KRT initiation) is not associated with worse post-KRT survival, and appears to be associated with lower mortality, directly challenges long-standing assumptions in nephrology. The authors’ proposal to reconsider time-based definitions of late referral and move toward a risk-based framework is conceptually sound and aligned with contemporary CKD management paradigms. However, this counterintuitive finding requires more cautious interpretation. The current manuscript risks implying a protective effect of late referral, which may be misleading given the retrospective design and the strong potential for residual confounding, selection bias, and reverse causality. The conclusions should be tempered to clearly distinguish association from causation.

2. Methodological limitations and potential bias

- Late referral is defined retrospectively based on time to KRT initiation. This definition inherently excludes patients who die before reaching KRT, introducing survivor bias. Also, patients referred early may represent a systematically sicker group with greater comorbidity burden, which itself drives mortality risk, independent of referral timing. This limitation should be more explicitly acknowledged, particularly when interpreting survival differences after KRT.

- Patients with higher comorbidity burden, more rapid decline, or complex disease are more likely to be referred earlier. This may explain the observed higher mortality within and more than 12 months in the “early referral” group. Although comorbidity burden is discussed descriptively, it is not adequately controlled for in the final multivariable model.

- The study includes only patients already referred to public nephrology clinics in Queensland. Patients managed exclusively in primary care or private settings are not represented.

As such, the findings may not be generalizable to all CKD populations or to healthcare systems outside Australia.

3. Data Analysis and Interpretation

- Please show the table of univariate results and the final Cox model.

- It is unclear why other clinically important variables (e.g., diabetes, socioeconomic status, Indigenous status, comorbidity score as a composite) were excluded beyond a univariate p-value threshold. This approach may lead to model under-adjustment and overestimation of the apparent effect of referral timing.

- Maybe include an additional adjusted model incorporating comorbidity score and socioeconomic status, given their central importance to the study’s conclusions.

- Clarify whether proportional hazards assumptions were tested.

4. Interpretation and Conclusions

- The conclusion that referral timing “may no longer be reliable” as a determinant of outcomes is provocative but somewhat overstated given the observational nature of the data.

- The evidence more strongly supports the conclusion that comorbidity burden and socioeconomic disadvantage are dominant predictors of mortality, rather than that referral timing is unimportant.

Minor comments

1. Abstract: The Results section should more clearly state that the apparent survival advantage in late referral is likely confounded by comorbidity burden.

- Consider adding adjusted variables included in the final Cox model with p-value from table 2.

2. Terminology: Ensure consistent use of “early,” “timely,” and “late” referral throughout the manuscript.

- Clarify whether “early referral” always refers to >12 months before KRT.

3. Figures and Tables:

The figure showing the distribution of deaths by socioeconomic status should be revised to allow comparison between early and late referral groups.

Table 2 should present both univariate and multivariable analyses.

Table 3, which compares early versus late referral, would benefit from the inclusion of p-values for key baseline differences.

4. Missing clinical variables

Acknowledge the absence of data on dialysis modality, vascular access type, and cause of death as important unmeasured confounders.

5. References

Minor inconsistencies in formatting and typographical errors (e.g., spacing, hyphenation) should be corrected.

Language and Style

Extensive English editing is required to improve clarity, cohesion, and overall readability.
