# Supplementary material for: Referral patterns in the CKD.QLD Registry: A call for revisiting the definition of late referral
Source: PLoS One. 2026 Aug 26;21(8):e0338001. doi: 10.1371/journal.pone.0338001 (PMC13514023; doi:10.1371/journal.pone.0338001)
Supplement: Supplementary file 3 [file pone.0338001.s005.docx]

Jeerath Phannajit, M.D, Ph.D.
Academic Editor
PLOS One

14 February 2026

**PLOS ONE Author’s response to reviews**

**Title**: Referral patterns in the CKD.QLD Registry: a call for revisiting the definition of late referral

Submission ID PONE-D-25-61252

Dear Dr Phannajit,

Title: Referral patterns in the CKD.QLD Registry: a call for revisiting the definition of late referral

We thank you and the reviewers for the valuable feedback which has strengthened the manuscript. In response to the feedback, changes have been made to the manuscript. We would like to make the following comments:

1. Upon revising our manuscript, we identified a major error in our data handling that required reanalysis. Specifically, we had incorrectly censored all participants at the follow‑up closure date of 30 June 2018. This is obviously incorrect for those who died within the study follow-up period. Correcting this error removed the statistical significance of some results but did not change the overall pattern: we continued to find no evidence of harm from late referral, and comorbidity score remained a stronger predictor of mortality among participants who commenced kidney replacement therapy than timing of referral.
2. We have revised the manuscript to accommodate the corrected analyses and subsequent new results, as well as in response to the Reviewers’ comments.
3. We have attached a copy of our point-by-point response to the comments
4. The discussion, limitations, and conclusion sections of the manuscript have been revamped by incorporating the suggestions given by the reviewers.

All changes made to the original submitted manuscript are shown with tracked changes in the revised manuscript.

We hope that this revised manuscript will now be acceptable for publication in PLOS ONE

Yours Sincerely,

Dr Clyson Mutatiri (corresponding author)

R**esponses to the Editors’ and the Reviewers’ Comments**

| **Editor(s)’ comments** | **Authors’ responses** |
| --- | --- |
| 1. **Methodological concerns**:   Please address all methodological concerns raised by both reviewers. You should prioritize minimizing the biases identified (specifically regarding the definition of "late referral" and variable selection). Where you cannot adjust the methodology, you must clearly state your rationale and explicitly discuss the re Language: | The methodological concerns raised by both reviewers have been carefully considered and addressed, as appropriate, with the manuscript revised accordingly. |
| 2. **Language:** As PLOS ONE does not copyedit accepted manuscripts, the language in the submission must be clear, correct, and unambiguous. I recommend reviewing the text carefully or seeking independent editorial help to ensure it meets these standards before resubmission. | Language and text used in the manuscript has been carefully reviewed and appropriate corrections have been made to meet PLOS ONE standards. |
| 3. Please ensure that your manuscript meets PLOS ONE's style requirements, including those for file naming. | The files have been renamed to comply with PLOS ONE style |
| 4. Your ethics statement should only appear in the Methods section of your manuscript. If your ethics statement is written in any section besides the Methods, please delete it from any other section. | We confirm, the ethics statement appears only in the methods section of our manuscript |
| 5. We notice that your supplementary figures are uploaded with the file type 'Figure'. Please amend the file type to 'Supporting Information'. Please ensure that each Supporting Information file has a legend listed in the manuscript after the references list. | The amendment has been done, and a legend has been created for each file, located in the manuscript after the reference list. |
| 6. Please include a separate caption for each figure in your manuscript. | Separate captions have been included for each figure |
| 7. If the reviewer comments include a recommendation to cite specific previously published works, please review and evaluate these publications to determine whether they are relevant and should be cited. There is no requirement to cite these works unless the editor has indicated otherwise. | No recommendations were made for specific citations |
| **Reviewers’ comments** |  |
| **Reviewer: 1** |  |
| **Major Comments**   1. “Late referral” is determined after follow-up information is known (KRT within 12 months), so it is not a baseline exposure available at the time of referral. Comparing post-KRT survival between “late” vs “early” referrals inherently conditions on surviving to KRT and on time accrued under nephrology care, introducing immortal time and selection bias. The surprising protective association (HR <12 months 0.46) is therefore highly likely to be artefactual rather than causal or clinically meaningful. | We agree with your comment on the definition of late referral, which currently uses the interval between a patient’s first nephrology clinic encounter and initiation of KRT in those who progress to end-stage kidney failure. As highlighted in our previous manuscript (1), this definition has important limitations. It excludes patients who are referred late but die before starting KRT, a group likely to differ clinically from those who survive to dialysis. It also overlooks many patients with advanced CKD who never require KRT and may have been appropriately managed without it. Furthermore, by focusing on the timing of dialysis initiation, this definition risks minimizing the broader objectives of early referral, including accurate diagnosis, slowing CKD progression, management of comorbidities (such as cardiovascular disease), and treatment of CKD-related complications. We were equally surprised by the seemingly protective association of “late referral” in our study, and although we agree that it is unlikely to be causal, this result reinforces our call for this definition to be revisited.  Reference: 1. Mutatiri C, Ratsch A, McGrail M, Venuthurupalli SK, Chennakesavan SK. Primary and specialist care interaction and referral patterns for individuals with chronic kidney disease: a narrative review. BMC nephrol. 2024;25(1):149. |
| 2. It is unclear whether the Cox model is anchored at registry enrolment, first nephrology visit, or KRT initiation—and whether deaths before KRT are included in the same model where “late referral” is defined only among those progressing to KRT. If the model includes the whole cohort, “late referral” cannot be treated as a fixed covariate without time-dependent handling; if restricted to KRT starters, the model must clearly define time zero at KRT start and adjust for baseline status at KRT start. | Sorry for any confusion from omitted key details. Our Cox model is anchored at KRT initiation and hence does not include deaths before KRT. Because the model is anchored at the start of KRT, baseline characteristics vary across the cohort despite the shared outcome of progressing to KRT.  We have amended our Methods section to ensure that this is now unambiguously clear to the reader. |
| 3. The manuscript’s own discussion suggests sicker/high-comorbidity patients were preferentially referred earlier. This strongly biases associations between “referral timing” and mortality against early referral. to resolve this (especially with crude comorbidity handling and variable selection based on univariate p-values). | We have acknowledged in our limitations section how the early referral of sicker patients with high comorbidity scores might have biased the observed associations between “referral timing” and mortality against early referral. |
| 4. The paper infers guideline-discordant or potentially “inappropriate” referrals based on eGFR/UACR thresholds, but acknowledges inability to determine referral reasons. Without referral indications (e.g., hematuria, refractory HTN, GN suspicion, rapid decline, electrolyte disorders), appropriateness cannot be concluded—only “did/did not meet specific thresholds.” | We agree. The manuscript has been revised and the terminology inferring guideline-discordant or potentially “inappropriate” referrals has been replaced with “did/did not meet specific thresholds”. |
| 5. KFRE analysis is presented as “predictive accuracy” without proper metrics Statements that eGFR threshold is “as effective as KFRE” are not supported by performance evaluation (discrimination, calibration, decision-curve analysis, sensitivity/specificity/PPV/NPV). Reporting proportions that progress to KRT is not “accuracy.” | The term accuracy has been dropped and the KFRE analysis has been limited to proportions that progressed to KRT. |
| 6. Late referral occurs in only 60 KRT patients with 15 deaths post-KRT (and only 3 deaths within 12 months). Any survival comparisons are statistically fragile and highly sensitive to residual confounding and model specification. | We have now acknowledged in our manuscript the fact that survival comparisons in our study would be statistically fragile and highly sensitive to residual confounding and model specification due to the small sample size of participants who were referred late, resulting in a small number of post-KRT deaths and hence the results should be interpreted with caution. |
| 7. Model building approach raises risk of overfitting and biased estimates Stepwise exclusion based on univariate p>0.2 is discouraged; it inflates type I error and biases coefficients. Pre-specified confounder sets or penalized approaches would be preferable. | A pre-specified set of confounders (age at KRT start, gender, Indigenous status, diabetes mellitus status, CVD, hypertension, CAD, primary renal disease, social status and composite comorbidity score) were used as confounder variables against the exposure variable of “late referral” |
| **Moderate concerns**   8. Comorbidity burden is dichotomized by simple count (0–2 vs ≥3) across 17 comorbidities, without weighting or validation (e.g., Charlson index). This may misclassify severity and leave major residual confounding. | The comorbidity scores have been recalculated using the modified and unweighted Charlson Comorbidity index. |
| 9. Missing data: UACR missing for ~31%; some UACR is imputed via equations (PCR/dipstick conversion). The impact on KFRE and staging is not quantified; no multiple imputation or sensitivity analyses are shown. | Thank you for your comment. We would like to note that the ~31% participants with missing values, either measured or converted, were not included in the KFRE equation. The missing data on these 31% of the participants has been acknowledged as a limitation of our study  For the participants who had urine dipstick and urine PCRs available, we used the alternative conversion method developed by Sumida et al to calculate the UACR rather than multiple imputation. However, this conversion method has significant limitations, and these have also been acknowledged in the manuscript (line 453 to 459). |
| 10. Generalizability: public nephrology clinics only; potential exclusion of private care pathways and different referral dynamics. | This has now been acknowledged in the manuscript |
| **Minor issues (examples)**   11. Clarify terminology: “referral date” vs “first nephrology visit” vs “registry enrolment” these are used interchangeably. | The distinction between “first nephrology visit” and “referral date” has been clarified (lines 38 to 40), and the term referral is now used consistently throughout the manuscript. However, Registry enrolment did not always occur at the time of referral, as some participants were enrolled from the group of patients who were already attending the clinics. This phrase is used separately from the other 2 phrases. |
| 12. Ensure consistent reporting of medians vs means and specify distributions used for tests. | All the results are now reported as means with corresponding distributions. |
| 13. Specify which KHA referral criteria (year/version) and justify chosen thresholds. | This has been rectified by adding paragraph 155 - 160 to the manuscript. |
| **Reviewer: 2** |  |
| 1. Overall significance and interpretation of the main finding - The finding that “late referral” (defined as ≤12 months before KRT initiation) is not associated with worse post-KRT survival, and appears to be associated with lower mortality, directly challenges long-standing assumptions in nephrology. The authors’ proposal to reconsider time-based definitions of late referral and move toward a risk-based framework is conceptually sound and aligned with contemporary CKD management paradigms. However, this counterintuitive finding requires more cautious interpretation. The current manuscript risks implying a protective effect of late referral, which may be misleading given the retrospective design and the strong potential for residual confounding, selection bias, and reverse causality. The conclusions should be tempered to clearly distinguish association from causation. | We agree that it is not our intent to imply that later-referral has a ‘protective effect’.  To reduce any confusion of such, conclusions have been revised to reflect the fact that timely referral remains central to improving CKD outcomes. |
| 2. Methodological limitations and potential bias - Late referral is defined retrospectively based on time to KRT initiation. This definition inherently excludes patients who die before reaching KRT, introducing survivor bias. Also, patients referred early may represent a systematically sicker group with greater comorbidity burden, which itself drives mortality risk, independent of referral timing. This limitation should be more explicitly acknowledged, particularly when interpreting survival differences after KRT. - Patients with higher comorbidity burden, more rapid decline, or complex disease are more likely to be referred earlier. This may explain the observed higher mortality within and more than 12 months in the “early referral” group.   Although comorbidity burden is discussed descriptively, it is not adequately controlled for in the final multivariable model. - The study includes only patients already referred to public nephrology clinics in Queensland. Patients managed exclusively in primary care or private settings are not represented. As such, the findings may not be generalizable to all CKD populations or to healthcare systems outside Australia. | The limitations section of the discussion has been revamped to reflect these limitations.  We have also taken on board the comment regarding comorbidity burden by using the modified, unweighted Charlson Comorbidity Score, which has previously been applied in dialysis populations to predict outcomes such as mortality and hospitalisation. Unlike the original Charlson Index which assigns weighted values (1 to 6) to different conditions, the unweighted version simply counts the number of qualifying chronic conditions, with each contributing a score of 1. Evidence suggests that this simpler count performs similarly to the weighted index in predicting long‑term mortality. Accordingly, we have updated our Methods section and revised our analyses to incorporate this improved comorbidity measure. |
| 3. Data Analysis and Interpretation - Please show the table of univariate results and the final Cox model. - It is unclear why other clinically important variables (e.g., diabetes, socioeconomic status, Indigenous status, comorbidity score as a composite) were excluded beyond a univariate p-value threshold. This approach may lead to model under-adjustment and overestimation of the apparent effect of referral timing. - Maybe include an additional adjusted model incorporating comorbidity score and socioeconomic status, given their central importance to the study’s conclusions. - Clarify whether proportional hazards assumptions were tested. | The univariate Cox model, with late referral as the predictor has now been added to the manuscript. The full multivariate model is also now included, together with the final model adjusted for comorbidity score and socioeconomic status. |
| 4. Interpretation and Conclusions - The conclusion that referral timing “may no longer be reliable” as a determinant of outcomes is provocative but somewhat overstated given the observational nature of the data. - The evidence more strongly supports the conclusion that comorbidity burden and socioeconomic disadvantage are dominant predictors of mortality, rather than that referral timing is unimportant. | We strongly agree with this comment. While our findings show that comorbidity burden and socioeconomic disadvantage are significant drivers of mortality, this does not mean that referral timing lacks importance. Rather, it suggests that these other factors seem to have a comparatively stronger influence on mortality. The conclusion has therefore been updated to clarify this point and to remove the phrase “may no longer be reliable.” |
| **Minor comments** 1. Abstract: The Results section should more clearly state that the apparent survival advantage in late referral is likely confounded by comorbidity burden. - Consider adding adjusted variables included in the final Cox model with p-value from table 2. | A sentence has been added to the results section of the abstract – “The absence of an observed adverse effect of late referral on KRT mortality in our study is likely explained by confounding from comorbidity burden” (lines 57-58).  Some effect sizes for key variables (taken from the MV Cox model) have been added into the Abstract-Results. |
| 2.Terminology: Ensure consistent use of “early,” “timely,” and “late” referral throughout the manuscript.  Clarify whether “early referral” always refers to >12 months before KRT | This has been done  The definition of early/late referral has been clarified |
| **Figures and Tables:** 3. The figure showing the distribution of deaths by socioeconomic status should be revised to allow comparison between early and late referral groups. | The figure has been replaced with a table (Table S4), which displays the distribution of death by referral timing and socioeconomic status much clearer. |
| Table 2 should present both univariate and multivariable analyses.  Table 3, which compares early versus late referral, would benefit from the inclusion of p-values for key baseline differences. | Table 3 has been revised to present both univariate and multivariate analyses.  p-values included for baseline variables in Table 4 |
| 4. Missing clinical variables Acknowledge the absence of data on dialysis modality, vascular access type, and cause of death as important unmeasured confounders. | This has now been fully acknowledged by adding lines 450 to 452 to the manuscript. |
| 5. References Minor inconsistencies in formatting and typographical errors (e.g., spacing, hyphenation) should be corrected. | The references have been thoroughly revised to address this concern. |
