# Supplementary material for: Referral patterns in the CKD.QLD Registry: A call for revisiting the definition of late referral
Source: PLoS One. 2026 Aug 26;21(8):e0338001. doi: 10.1371/journal.pone.0338001 (PMC13514023; doi:10.1371/journal.pone.0338001)
Supplement: Supplementary file 4 [file pone.0338001.s006.docx]

| **Editor(s)’ comments** | Authors’ responses |
| --- | --- |
| 1. **Analytical mitigation for survivor bias and selection bias:**   Both reviewers independently identify the lack of any analytical strategy to mitigate immortal time bias, survivor bias, and competing risks as the central methodological weakness. The definition of late referral based on time to KRT conditions on surviving to KRT, and the survival analysis anchored at KRT initiation further restricts the population to a selected subgroup. Acknowledgement of these limitations in the text, while appreciated, is insufficient on its own. We request that the authors attempt at least one complementary analysis to address this — for example, a competing-risks regression (Fine-Gray model) treating pre-KRT death as a competing event, or a landmark analysis. If the authors determine that such analyses are not feasible given the data structure or sample size, they must provide a clear methodological explanation of why this is the case and substantially strengthen the cautionary language in both the Abstract and the Discussion. **At minimum, the Discussion should include an explicit statement that the absence of a statistically significant association cannot be interpreted as evidence that late referral is safe, and that the study is underpowered to exclude clinically meaningful harm**. Authors should also acknowledge that restricting analysis to KRT starters may introduce collider bias if referral timing and comorbidity burden both determine who reaches KRT — this is not currently mentioned in the Limitations. | Thank you for these important methodological comments. We agree that our original analysis, restricted to patients who survived to KRT initiation (n = 513), was subject to immortal time bias and survivorship selection bias, as it excluded the large number of individuals who died after initial referral but prior to starting KRT.  In response to your request, we conducted a complementary analysis designed to address these limitations. Specifically, we applied a left-truncated (delayed entry) Fine-Gray subdistribution hazards model to the full referred cohort (n = 3,775).  To enable this, our data were restructured as follows:   1. **Time origin**: Follow-up was defined from the date of initial nephrology referral for all participants. This allowed inclusion of all study data, including deaths occurring before KRT (n = 598, 15.8%) as competing risk events. 2. **Delayed entry**: Participants who initiated KRT (n = 513) entered the primary risk set at the date of KRT initiation. This approach maintains a focus on post-KRT outcomes while avoiding immortal time bias. 3. **Follow-up period**: Follow-up time was left-truncated at KRT initiation and censored at death or the end of the study   We would also like to clarify how the primary exposure variable, “late referral” was defined across the two models.  In the primary Cox model, analysis was restricted to patients who initiated KRT (n = 513). In this group, late referral was defined as a binary variable (within 12 months vs. >12 months before KRT), as all participants had a known KRT start date.  In contrast, for the analysis of the full cohort (n = 3,775) using the Fine-Gray model, a binary classification was not appropriate. Participants who died before KRT (n = 598) did not initiate treatment and therefore could not be classified as early or late referrals. Applying a binary definition in this setting would introduce bias. Instead, we used a continuous measure of exposure, defined as the total time under nephrology care from initial referral (in months). This approach allowed inclusion of all participants and provided a consistent and unbiased representation of referral timing across the full cohort.  Although the results from the adjusted competing risks model (reported in the Methods, Results, and Table 4) support our previous overall conclusions, some differences emerged.  Notably, we found that longer duration of pre-KRT care was associated with a higher risk of post-KRT mortality (SHR 1.0038 per additional month; 95% CI 1.0012–1.0064; p = 0.004). This corresponds to a 0.38% relative increase in hazard per month, or approximately 4.7% over one year of care. Although the effect was small, it was statistically significant.  Baseline clinical risk remained a strong predictor of post-KRT mortality. Each one-unit increase in comorbidity score was associated with more than a twofold increase in mortality risk (SHR 2.113, 95% CI 1.792-2.493; p < 0.001), and pre-existing cardiovascular disease was also independently associated with higher risk (SHR 1.910; p = 0.004). |
| 2. **Clarify the Cox model population and time zero in the Methods:**   The Methods section currently states that the Cox model examined "associations between time of referral and the outcomes of death and KRT initiation" without specifying that the survival analysis was restricted to KRT starters (n=513) with time zero at KRT initiation. Please add an explicit sentence confirming this, for example: "Survival analysis was restricted to participants who commenced KRT (n=513), with time zero defined as the date of KRT initiation; deaths occurring prior to KRT were not included in this analysis." | Thank you for this important comment. We have revised the Methods section to clearly define the study population and the baseline time points used in the primary Cox model. |
| 3. **Proportional hazards assumption:**   The manuscript does not state whether the proportional hazards assumption was tested. This is a standard requirement for Cox regression reporting. Please add a sentence in the Methods confirming whether the assumption was tested (e.g. using Schoenfeld residuals) and reporting the result. | Thank you for the comment. We agree that assessment of the proportional hazards (PH) assumption is an important reporting requirement. We have revised the Methods to state that this assumption was evaluated using Schoenfeld residuals, with no evidence of violation (p > 0.05). |
| 4. **Subgroup analyses at shorter referral thresholds:**   Reviewers note that the 12-month definition of late referral is broader than most prior studies, which typically use cut-offs of 3–6 months, and that this may dilute the observed association with adverse outcomes. We ask the authors to either (a) perform additional analyses using 3-month and/or 6-month thresholds to improve comparability with the existing literature, or (b) explicitly state in the Methods section why meaningful analysis at shorter thresholds is not feasible — for example, due to the very small number of participants who would qualify as "late referral" under stricter definitions, which would render any comparison statistically unreliable. Either response is acceptable provided it is clearly justified. | Thank you for this important comment on temporal definitions. We addressed this in two ways.  First, we clarified in the Methods our clinical rationale for selecting a 12-month threshold to indicate “late referral”, reflecting the time typically required for adequate pre-dialysis preparation, including permanent vascular access.  Second, rather than conducting a small subgroup analysis, we performed a multivariable left-truncated Fine-Gray competing risks sensitivity analysis in the full cohort (n = 3,775). We modelled the risk of post-KRT mortality across several thresholds (1, 3, 6, and 12 months of pre-KRT care). As shown in Figure S3, the resulting cumulative incidence curves were similar, indicating no meaningful differences in risk prediction across these thresholds after accounting for competing risks and comorbidities. |
| 5. **Correct the confidence interval error in Table 3:**   The Social Status row in Table 3 reports a 95% confidence interval of [1.75–1.58], in which the lower bound exceeds the upper bound. This is a transcription error and must be corrected. | Thank you for identifying this transcription error. The error has now been corrected. |
| 6. **Correct the Methods description of model-building:**   Table 2 correctly presents a full pre-specified model including all clinically important covariates. However, the Methods text still states that "the final model excluded variables with little univariate significance (p-value of >0.2)." This contradicts what is shown in Table 2. Please revise the Methods text to accurately describe the approach used — namely, that a pre-specified confounder set was retained in the model regardless of univariate significance. | Thank you for the comment and recommendation. The description of the Cox model has been revised, incorporating the recommendation. |
| 7. **The Conclusion states that the eGFR threshold "demonstrated similar prediction" to the KFRE.**   As noted by Reviewer 1, no formal measures of predictive performance (discrimination, calibration, or decision-curve analysis) were conducted, and this phrasing implies a comparative inferential claim that is not supported. Please revise to descriptive language, for example: "identified similar proportions of participants who progressed to KRT." | Thank you for this correction. We agree that assessing the predictive performance of a risk model requires measures of discrimination (such as C-indices or AUC) and calibration, which were not evaluated in our study. We have therefore revised the descriptive language as explained in more detail in our response to Reviewer 1’s comment number 4. |
| 8. **The rationale for the 12-month cut-off (elimination of vascular access as a confound) currently appears only in the Discussion.**   A brief statement of this justification should also be included in the Methods section at the point where late referral is defined. | Thank you for the comment and recommendation. A statement of justification has now been added to the Methods section of the body of the manuscript under Outcomes and variables. |
| 9. **Reference 18 contains "Octboer" (should be "October") and Reference 34 contains "dialysis access" (should be "dialysis access").**   Please correct these and check the reference list for any remaining formatting inconsistencies. | Thank you for the correction. The typographical errors have been addressed. Reference 34 has been re-numbered as reference 39, while reference 18 is no longer cited in the manuscript. |
| 10. **Discussion framing — absence of evidence:**   Although the revised conclusion is more measured than the original, some language in the Discussion (for example, describing the non-significant Kaplan-Meier trend as warranting "clinical consideration") may still give readers the impression that the data support a clinically meaningful signal. We recommend adding a clear statement such as: "Absence of a statistically significant association should not be interpreted as evidence of absence of harm; this study was not adequately powered to exclude a clinically important mortality difference between referral groups." | Thank you for this important suggestion. We have revised the Discussion to clarify that the non-significant finding in our primary analysis should not be interpreted as evidence of no association, given limited statistical power for this comparison. |
| 11. **Minor inconsistencies in the use of "referral date" versus "first nephrology visit" persist in the Abstract.**   Please review and ensure uniform terminology throughout**.** | We thank you for this comment. We have revised the terminology to ensure consistency in the use of referral date. |
| 12. **Table 4:**   The UACR mean and SD for the early referral group are presented as "102.0 (145.7)" without the "SD" label, inconsistent with the column header and other rows. Please standardise | Thank you for the comment. We have standardised the labels. |
| **Reviewers’ comments** |  |
| **Reviewer: 1** |  |
| **Major Comments**   1. This manuscript addresses an important and clinically relevant question regarding referral patterns in chronic kidney disease and their relationship with patient outcomes. The attempt to re-examine conventional time-based definitions of “late referral” and to move toward a more risk-based conceptual framework is thoughtful and aligns with evolving perspectives in CKD care. The authors have clearly made a genuine effort to respond to prior reviewer comments, particularly by clarifying terminology, moderating some interpretations, and expanding the discussion of limitations. However, despite these improvements, several important methodological concerns remain insufficiently addressed, and these limit confidence in the validity and interpretation of the findings. Most notably, the definition of “late referral” continues to be derived retrospectively based on time to KRT initiation. This approach inherently conditions on future events and excludes patients who die prior to reaching KRT, thereby introducing selection and immortal time bias. Although the authors acknowledge these limitations, the current revision does not include any analytical strategy to mitigate them. As a result, the primary exposure remains difficult to interpret in a causal framework, and the observed associations may be substantially biased. Related to this, the clarification that the survival analysis is anchored at the time of KRT initiation is helpful but raises additional concerns. Restricting the analysis to patients who survive to KRT introduces a selected population and may lead to collider bias, particularly if referral timing and comorbidity burden both influence progression to KRT. The manuscript would benefit from a more explicit justification of this design choice, along with consideration of complementary analyses that account for the pre-KRT period or competing risks such as death prior to KRT. At minimum, the implications of this conditioning should be more clearly articulated. | Thank you for the comment. We agree with this important methodological point. Restricting survival analyses to patients who reach KRT can introduce immortal time bias and potential selection-related biases.  To address this, we conducted a complementary multivariable competing risks analysis using a left-truncated Fine-Gray model in the full cohort (n = 3,775). Time zero was defined as the date of initial nephrology referral, allowing inclusion of deaths prior to KRT (n = 598) as competing events. Patients who initiated KRT entered the risk set at that time, maintaining a focus on post-KRT outcomes.  Methods, Results, and Discussion to reflect this approach and its findings. |
| 2. **Concerns regarding confounding also remain.**   While the inclusion of a comorbidity score represents an improvement, the use of an unweighted Charlson index may not adequately capture differences in disease severity, and residual confounding is likely substantial. In addition, there remains some ambiguity regarding the model-building strategy, particularly whether variables were selected based on prior knowledge or data-driven approaches. Clarifying this point and ensuring that key clinical confounders are consistently included in the primary model would strengthen the analysis and improve interpretability. | Thank you for this important comment on confounding. We acknowledge that an unweighted comorbidity count may not fully capture differences in disease severity, and we have clarified this limitation in the Discussion.  Covariates were selected a priori based on established clinical knowledge and prior literature on mortality risk in ESKD, rather than using automated selection methods. To reduce residual confounding, our competing-risks model also included key high-risk conditions, diabetes mellitus, hypertension, cardiovascular disease, and coronary artery disease, alongside the overall comorbidity score. |
| 3. The interpretation of the findings has been partially moderated, which is appreciated. Nevertheless, some statements still risk overemphasizing the apparent lack of harm, or even potential benefit, associated with later referral. Given the observational design and the multiple sources of bias discussed above, it is important that the conclusions consistently emphasize uncertainty and avoid suggesting that referral timing is unimportant. A more cautious framing that highlights the role of confounding, selection effects, and competing risks would be more appropriate. | Thank you for this important comment. We have revised the Discussion and Limitations to emphasise the observational design and the potential for residual confounding and selection effects.  We have also added clarification on missing data and generalisability. Missing UACR data were handled using listwise deletion in specific models, which may introduce bias, especially if missing values correlate with underlying disease severity. We also note that the cohort was drawn from public nephrology clinics, which may limit generalisability to other healthcare settings. |
| 4. The analysis of KFRE also remains limited. Although the terminology has been adjusted, the current presentation does not constitute a true evaluation of predictive performance. Without appropriate measures of discrimination or calibration, comparisons between KFRE and eGFR-based thresholds should either be removed or clearly described as descriptive rather than inferential. | Thank you for this correction. We agree that assessing the predictive performance of a risk model requires measures of discrimination (such as C-indices or AUC) and calibration, which were not evaluated in our study. Following this suggestion, we have retained the KFRE comparison because of its relevance to our overall conceptual framework. However, we have revised the Methods, Results, Discussion, and Limitations sections to clearly present these analyses as descriptive rather than inferential. We have also added the lack of formal discrimination and calibration assessment as a study limitation. |
| 5. **Finally, several additional issues warrant further clarification:**   The relatively small number of late referral patients and outcome events raises concerns about statistical stability, and this should be more explicitly addressed, ideally with additional sensitivity analyses or stronger caution in interpretation. The handling of missing data, particularly for UACR, also remains suboptimal and may introduce bias; a clearer explanation of the analytical approach and its implications is needed. Issues of generalizability, given the restriction to public nephrology clinics, and the potential limitations of the comorbidity measure should be further elaborated in the discussion. | Thank you for these comments. We have revised the manuscript to improve transparency.  We have added clarification regarding statistical power, noting the small number of late referral cases (n = 60) in the primary analysis meaning that our study was likely underpowered. We have also clarified our handling of missing data, specifically that urine albumin-to-creatinine ratio (UACR) was managed using listwise deletion in descriptive analyses, which may introduce bias if data were not missing at random.  In addition, we have expanded the Limitations section to address generalisability and measurement issues. Our data are derived from public nephrology clinics, which may limit applicability to other healthcare settings. We also note that the comorbidity score used in this study is an unweighted summary measure and may not fully capture differences in disease severity. |
| **Reviewer: 2** |  |
| Major Comments   1. **Overall significance and interpretation of the main finding**   The finding that “late referral” is not associated with worse outcomes—and may even appear protective—is counterintuitive and challenges established evidence. The manuscript risks overstating this finding.  The observed association is likely influenced by residual confounding, selection bias, and reverse causality. This study also shows an imbalance between early referrals (448 cases) and late referrals (60 cases).  The definition of late referral used in this study (initiation of KRT within 12 months of the first nephrology visit) is broader than that in most prior studies, which commonly apply cut-offs of 3–6 months. While this approach may improve sensitivity in capturing delayed care, it could also dilute the association with adverse outcomes. Importantly, a large body of evidence has consistently demonstrated that late referral is associated with increased mortality, likely due to inadequate pre-dialysis preparation and higher rates of unplanned dialysis initiation.  Therefore, the authors should justify their chosen definition and consider additional subgroup analyses using shorter cut-offs to enhance comparability with existing literature such as in previous study, there was significantly increased overall mortality in the late referral group as compared with the early referral group (relative risk 1.99; 95% confidence interval [CI], 1.66 to 2.39, P <.0001). | Thank you for this important comment. We agree that, in traditional survival analyses, using a 12‑month threshold may reduce sensitivity, and that our primary Cox model was limited by imbalance between early and late referral groups.  To address this, we performed a sensitivity analysis using the full cohort (n = 3,775) and a multivariable competing risks framework, modelling time under care continuously. From this model, we estimated post-KRT cumulative incidence at 1, 3, 6, and 12 months of pre-KRT care (Supplementary Figure 3).  The resulting curves were closely aligned, indicating no meaningful differences in risk across these thresholds after accounting for competing risks and baseline comorbidity. This suggests that the findings are not driven by the choice of a 12‑month definition. Rather, they indicate that fixed time-based thresholds (e.g. 3, 6, or 12 months) may have limited ability to distinguish clinical risk once underlying patient characteristics are considered.  We have revised the manuscript to reflect these findings and to present a more cautious interpretation, emphasising the limitations of time-based definitions and the potential value of risk-based approaches. |
| 2. **Methodological limitations and potential bias**   Late referral is defined based on time to KRT, excluding patients who die before KRT and introducing survivor bias.  Patients referred earlier are likely sicker (higher comorbidity), creating confounding by indication.  Recommendation: Expand discussion of bias and clarify its impact on findings. Acknowledge limited generalisability. | Thank you for the comment. We agree with this important point. Observational studies of referral timing are prone to both survivor bias and confounding by indication, which can influence results in opposite directions.  To address these issues, we applied a left-truncated Fine-Gray model that included deaths occurring before KRT (n = 598) as competing events, reducing survivor bias. In addition, our multivariable models adjusted for key clinical conditions, including cardiovascular disease, hypertension, diabetes, and overall comorbidity burden, to limit confounding by indication.  We have expanded the Discussion to clarify how these sources of bias may affect interpretation and to emphasise the need for cautious interpretation of observational findings. |
| 3. **Data analysis and internal consistency**   There are inconsistencies in reported results, including hazard ratios and significance. Subgroup analysis using cut-offs of 3 or 6 months may be needed.  Variable selection based on univariate p-values may lead to under-adjustment.  Recommendation: Clearly define models, include full tables, and ensure consistency across manuscript. | Thank you for this comment. We have revised the manuscript to ensure consistency of all numerical values across the text, abstract, and figures.  We also agree that selecting covariates based solely on univariate p-values can lead to inadequate adjustment and residual confounding. Therefore, we did not use automated selection methods. Instead, all multivariable analysis models were specified a priori, with inclusion of established demographic, cardiovascular, and metabolic confounders (age, sex, Indigenous status, diabetes, cardiovascular disease, hypertension, coronary artery disease, social status, and comorbidity score) regardless of statistical significance. Full results for both models are provided in the main text. |
| 4. **Interpretation of results and conclusions**   The conclusion that referral timing may no longer be reliable is overstated.  Recommendation: Emphasise uncertainty and highlight comorbidity and socioeconomic factors as key predictors. . | Thank you for this constructive suggestion. We have revised the abstract, results, and discussion to ensure a more cautious interpretation of our findings, consistent with the limitations of an observational study.  We now emphasise the uncertainty surrounding our results and avoid overstating conclusions. While referral timing remains independently associated with mortality risk, its effect is modest compared with baseline clinical and social factors, including comorbidity burden (SHR 2.113; p < 0.001), pre-existing cardiovascular disease (SHR 1.910; p = 0.004), and Indigenous status (SHR 1.931; p = 0.007). |
| 5. **Definition of late referral**   The 12-month definition is not sufficiently justified. Alternative cut-offs of 3 or 6 months may be needed. Recommendation: Justify threshold or include sensitivity analyses. | Thank you for this suggestion. We have expanded the justification for the 12-month threshold in the Methods. Clinically, this period reflects the time typically required for preparation before dialysis, including vascular access planning and patient education.  To examine shorter intervals without introducing instability from small subgroup sizes, we used the full cohort (n = 3,775) and modelled time under care continuously in a multivariable left-truncated Fine–Gray framework. Based on this model, we estimated post-KRT cumulative incidence at 1, 3, 6, and 12 months of pre-KRT care (Figure S3). The resulting curves were similar, indicating that risk estimates did not differ meaningfully across these time points, supporting the robustness of our primary findings. |
| 6. Role of KFRE and risk-based referral KFRE analysis is underdeveloped and not integrated into main models.  Recommendation: Strengthen analysis or moderate conclusions. | Thank you for this important suggestion. We have revised our analysis of the KFRE. As formal measures of discrimination and calibration were not performed, all KFRE-related analyses are now clearly presented as descriptive. We have removed language suggesting predictive performance and moderated the conclusions accordingly. |
| 7. **Minor Comments** i. Abstract: Clarify confounding by comorbidity. ii. Terminology: Ensure consistency of referral definitions. iii. Tables/Figures: Improve formatting and include p-values. iv. Missing variables: Acknowledge absence of modality, access, cause of death. v. Data quality: Correct typographical and formatting errors | Thank you for these comments. We have systematically reviewed and revised the listed points. |
