# Supplementary material for: Referral patterns in the CKD.QLD Registry: A call for revisiting the definition of late referral
Source: PLoS One. 2026 Aug 26;21(8):e0338001. doi: 10.1371/journal.pone.0338001 (PMC13514023; doi:10.1371/journal.pone.0338001)
Supplement: Supplementary file 5 [file pone.0338001.s007.docx]

| **Editor(s)’ comments** | Authors’ responses |
| --- | --- |
| **Please revise the manuscript to address the following points:** |  |
| 1. Please check and harmonise the reported effect estimates for late referral across the abstract, main text, and tables. The abstract reports that late referral was not associated with post-KRT mortality in the restricted Cox model, with adjusted HR 0.91, p = 0.74. However, Table 2 reports late referral HR 0.93, 95% CI 0.53–1.62, p = 0.80 in the multivariable model, while Table 3 reports late referral HR 0.82, 95% CI 0.47–1.41, p = 0.42. These may represent different models, but this is not clearly labelled. Please clarify which estimate is the primary adjusted Cox estimate, ensure that the same estimate is reported consistently, and clearly label any secondary or sensitivity models. | We thank the Editor for identifying this inconsistency. Following a comprehensive review of the manuscript, we determined that the discrepancy arose because different models were reported in different sections without sufficient explanation. Specifically, the Abstract and Results section referred to the final fully adjusted Cox proportional hazards model, whereas one of the tables contained estimates from an intermediate model and another table presented a secondary model including only selected covariates (old Table 3). This distinction was not sufficiently clear in the original submission.  To address this issue, we have:   - Deleted Table 3, which is not referenced in the main text. - Renamed Table 2 **“Univariate and Multivariable Cox Regression Analyses”** to improve clarity and accurately represent the data presented, including both univariate analyses and the fully adjusted multivariable Cox proportional hazards model. - Harmonised reporting throughout the Abstract, Results, Discussion, and tables so that the same primary adjusted estimate for late referral is presented consistently. - Revised the numbering of the tables and table headings.   **Revised Text**  **Abstract (Results):**  " In a fully adjusted Cox proportional hazards model restricted to the 513 participants who progressed to KRT, late referral was not associated with post-KRT mortality (HR 0.91; 95% CI 0.52-1.59; p = 0.74)."  **Results section:**  " When the comorbidity score was added, this estimate further decreased to 9% (HR 0.91; p = 0.74; CI 0.52–1.59)."  **Table Heading:**  "Table 2: **Univariate and multivariable Cox regression analyses** (N = 513)." |
| 2. Please correct the interpretation of the Fine–Gray competing-risks model. Table 4 reports time_under_care SHR 1.004 per month, 95% CI 1.001–1.006, p = 0.004. This indicates a small increase in the subdistribution hazard of post-KRT mortality per additional month of pre-KRT care, not a reduction. Therefore, the statement in the Conclusions that “longer pre-KRT nephrology care was associated with a modest reduction in post-KRT mortality” is inconsistent with the reported result and should be revised. Similar wording should also be checked and corrected in the abstract, Results, and Discussion. (also properly labeling the time_under_care). | We thank the Editor for identifying this important issue. We agree that the previous wording was inconsistent with the calculated results of the subdistribution hazard ratio. The Fine-Gray competing-risks model estimated a SHR of 1.004 per month (95% CI 1.001–1.006; p = 0.004), indicating a statistically significant but clinically small increase in the subdistribution hazard of post-KRT mortality associated with longer duration of pre-KRT care.  We have revised the Abstract, Results, Discussion, and Conclusions to ensure that the direction of the effect is described accurately and consistently. We have also replaced the variable label *time_under_care* with the more descriptive term ***duration of pre-KRT nephrology care (time under care)*** throughout the manuscript.  **Revised Text**  **Methods/Table 3:**  "Duration of pre-KRT nephrology care (time under care)."  **Abstract (Results):**  "By contrast, in a fully adjusted Fine-Gray model, longer pre-KRT care showed a statistically significant but clinically small effect on post-KRT survival (SHR 1.004 per additional month; 95% CI 1.001–1.006; p = 0.004)."  **Discussion:**  "In this framework, longer pre-KRT care was associated with a statistically significant but clinically small increase in post-KRT mortality risk (SHR 1.0038 per additional month; 95% CI 1.0012–1.0064; p = 0.004). This corresponds to a 0.38% relative increase in hazard per month, or approximately 4.7% over one year of care."  **Conclusions:**  "After accounting for pre-dialysis mortality to minimise survival bias, longer pre-KRT nephrology care was associated with a statistically significant but clinically small increase in post-KRT mortality risk." |
| 3. Please soften remaining policy or clinical implications that could be interpreted as supporting delayed referral or selective non-referral. The study supports the potential value of risk-stratified referral approaches, but it does not directly test the safety or effectiveness of delayed referral, non-referral, or primary-care-only management. Statements recommending changes to referral practice should be framed cautiously and as hypothesis-generating. | We appreciate this important observation and agree that our study should not be interpreted as evidence supporting delayed referral, non-referral, or primary-care-only management of CKD. As our study was observational and did not directly evaluate alternative referral strategies, causal inferences regarding the safety or effectiveness of delayed referral cannot be made.  Accordingly, we have revised the Discussion, the section entitled *The case for a new definition of late referral: A risk-based approach*, and the Conclusions to ensure that all clinical implications are framed cautiously. Recommendations have been reworded as exploratory and hypothesis-generating rather than prescriptive, and we now emphasise the need for prospective evaluation before changes to referral pathways can be recommended.  **Revised Text**  **Discussion:**  "Our findings should not be interpreted as evidence supporting delayed referral. Until such evidence is available, risk-based approaches should be considered a promising area for further investigation rather than a basis for changing current referral practices."  **Risk-based referral section:**  "Future prospective studies are needed to determine whether incorporating validated risk-prediction tools, such as the KFRE, at the time of referral can improve referral prioritisation and resource allocation while maintaining patient safety and equitable access to specialist care."  **Conclusions:**  "These findings suggest that retrospective, time-based definitions of referral timeliness have limited prognostic value in this cohort. However, the observational nature of this study precludes conclusions regarding the safety or effectiveness of delayed referral strategies or alternative referral pathways." |
