# Supplementary material for: Referral patterns in the CKD.QLD Registry: A call for revisiting the definition of late referral
Source: PLoS One. 2026 Aug 26;21(8):e0338001. doi: 10.1371/journal.pone.0338001 (PMC13514023; doi:10.1371/journal.pone.0338001)
Supplement: Peer review history

# Peer review: Decision Letter 0

**DOI:** 10.1371/journal.pone.0338001.r001

**Authors:** Jeerath Phannajit

5 Jan 2026

Dear Dr. Mutatiri,

Thank you for submitting your manuscript to PLOS ONE. After careful consideration, we feel that it has merit but does not fully meet PLOS ONE’s publication criteria as it currently stands. Therefore, we invite you to submit a revised version of the manuscript that addresses the points raised during the review process.

==============================

1. **Methodological concerns** : Please address all methodological concerns raised by both reviewers. You should prioritize minimizing the biases identified (specifically regarding the definition of "late referral" and variable selection). Where you cannot adjust the methodology, you must clearly state your rationale and explicitly discuss the reLanguage:
2. **Language:**  As PLOS ONE does not copyedit accepted manuscripts, the language in the submission must be clear, correct, and unambiguous. I recommend reviewing the text carefully or seeking independent editorial help to ensure it meets these standards before resubmission.sulting limitations in the manuscript.

Please submit your revised manuscript by Feb 19 2026 11:59PM. If you will need more time than this to complete your revisions, please reply to this message or contact the journal office at plosone@plos.org. When you're ready to submit your revision, log on to https://www.editorialmanager.com/pone/ and select the 'Submissions Needing Revision' folder to locate your manuscript file.

- A letter that responds to each point raised by the academic editor and reviewer(s). You should upload this letter as a separate file labeled 'Response to Reviewers'.
- A marked-up copy of your manuscript that highlights changes made to the original version. You should upload this as a separate file labeled 'Revised Manuscript with Track Changes'.
- An unmarked version of your revised paper without tracked changes. You should upload this as a separate file labeled 'Manuscript'.

If you would like to make changes to your financial disclosure, please include your updated statement in your cover letter. Guidelines for resubmitting your figure files are available below the reviewer comments at the end of this letter.

If applicable, we recommend that you deposit your laboratory protocols in protocols.io to enhance the reproducibility of your results. Protocols.io assigns your protocol its own identifier (DOI) so that it can be cited independently in the future. For instructions see: https://journals.plos.org/plosone/s/submission-guidelines#loc-laboratory-protocols. Additionally, PLOS ONE offers an option for publishing peer-reviewed Lab Protocol articles, which describe protocols hosted on protocols.io. Read more information on sharing protocols at https://plos.org/protocols?utm_medium=editorial-email&utm_source=authorletters&utm_campaign=protocols.

We look forward to receiving your revised manuscript.

Kind regards,

Jeerath Phannajit, M.D, Ph.D.

Academic Editor

PLOS One

Journal Requirements:

When submitting your revision, we need you to address these additional requirements.

1. Please ensure that your manuscript meets PLOS ONE's style requirements, including those for file naming. The PLOS ONE style templates can be found at https://journals.plos.org/plosone/s/file?id=wjVg/PLOSOne_formatting_sample_main_body.pdf and https://journals.plos.org/plosone/s/file?id=ba62/PLOSOne_formatting_sample_title_authors_affiliations.pdf

2. Your ethics statement should only appear in the Methods section of your manuscript. If your ethics statement is written in any section besides the Methods, please delete it from any other section.

3. We notice that your supplementary figures are uploaded with the file type 'Figure'. Please amend the file type to 'Supporting Information'. Please ensure that each Supporting Information file has a legend listed in the manuscript after the references list.

4. Please include a separate caption for each figure in your manuscript.

5. If the reviewer comments include a recommendation to cite specific previously published works, please review and evaluate these publications to determine whether they are relevant and should be cited. There is no requirement to cite these works unless the editor has indicated otherwise.

[Note: HTML markup is below. Please do not edit.]

Reviewers' comments:

Reviewer's Responses to Questions

**Comments to the Author**

1. Is the manuscript technically sound, and do the data support the conclusions?

Reviewer #1: Yes

Reviewer #2: Partly

**********

2. Has the statistical analysis been performed appropriately and rigorously?

Reviewer #1: Yes

Reviewer #2: N/A

**********

3. Have the authors made all data underlying the findings in their manuscript fully available?

The PLOS Data policy

Reviewer #1: Yes

Reviewer #2: Yes

**********

4. Is the manuscript presented in an intelligible fashion and written in standard English?

Reviewer #1: Yes

Reviewer #2: Yes

**********

Reviewer #1: This retrospective cohort study using the CKD.QLD Registry examines “timing” and “appropriateness” of nephrology referrals and links these to KRT initiation and mortality. The topic is important, and the dataset is potentially valuable. However, the central analytic construct “late referral” is defined retrospectively using future information (KRT within 12 months of first nephrology visit), and the current design/analysis creates major risks of immortal time/lead-time bias, selection (collider) bias, and confounding by indication, leading to results that are difficult to interpret

Major concerns

1. “Late referral” is determined after follow-up information is known (KRT within 12 months), so it is not a baseline exposure available at the time of referral. Comparing post-KRT survival between “late” vs “early” referrals inherently conditions on surviving to KRT and on time accrued under nephrology care, introducing immortal time and selection bias. The surprising protective association (HR <12 months 0.46) is therefore highly likely to be artefactual rather than causal or clinically meaningful.

2. It is unclear whether the Cox model is anchored at registry enrolment, first nephrology visit, or KRT initiation—and whether deaths before KRT are included in the same model where “late referral” is defined only among those progressing to KRT. If the model includes the whole cohort, “late referral” cannot be treated as a fixed covariate without time-dependent handling; if restricted to KRT starters, the model must clearly define time zero at KRT start and adjust for baseline status at KRT start.

3. The manuscript’s own discussion suggests sicker/high-comorbidity patients were preferentially referred earlier. This strongly biases associations between “referral timing” and mortality against early referral. to resolve this (especially with crude comorbidity handling and variable selection based on univariate p-values).

4. The paper infers guideline-discordant or potentially “inappropriate” referrals based on eGFR/UACR thresholds, but acknowledges inability to determine referral reasons. Without referral indications (e.g., hematuria, refractory HTN, GN suspicion, rapid decline, electrolyte disorders), appropriateness cannot be concluded—only “did/did not meet specific thresholds.”

5. KFRE analysis is presented as “predictive accuracy” without proper metrics

Statements that eGFR threshold is “as effective as KFRE” are not supported by performance evaluation (discrimination, calibration, decision-curve analysis, sensitivity/specificity/PPV/NPV). Reporting proportions that progress to KRT is not “accuracy.”

6.Late referral occurs in only 60 KRT patients with 15 deaths post-KRT (and only 3 deaths within 12 months). Any survival comparisons are statistically fragile and highly sensitive to residual confounding and model specification.

Model building approach raises risk of overfitting and biased estimates

Stepwise exclusion based on univariate p>0.2 is discouraged; it inflates type I error and biases coefficients. Pre-specified confounder sets or penalized approaches would be preferable.

Moderate concerns

1. Comorbidity burden is dichotomized by simple count (0–2 vs ≥3) across 17 comorbidities, without weighting or validation (e.g., Charlson index). This may misclassify severity and leave major residual confounding.

Missing data: UACR missing for ~31%; some UACR is imputed via equations (PCR/dipstick conversion). The impact on KFRE and staging is not quantified; no multiple imputation or sensitivity analyses are shown.

Generalizability: public nephrology clinics only; potential exclusion of private care pathways and different referral dynamics.

Minor issues (examples)

1.Clarify terminology: “referral date” vs “first nephrology visit” vs “registry enrolment” these are used interchangeably.

2.Ensure consistent reporting of medians vs means and specify distributions used for tests.

3.Specify which KHA referral criteria (year/version) and justify chosen thresholds.

Reviewer #2: Suggestions for major revisions

Review Comments to the Author

Issues that need to be addressed include the following:

Major Comments

1. Overall significance and interpretation of the main finding

- The finding that “late referral” (defined as ≤12 months before KRT initiation) is not associated with worse post-KRT survival, and appears to be associated with lower mortality, directly challenges long-standing assumptions in nephrology. The authors’ proposal to reconsider time-based definitions of late referral and move toward a risk-based framework is conceptually sound and aligned with contemporary CKD management paradigms. However, this counterintuitive finding requires more cautious interpretation. The current manuscript risks implying a protective effect of late referral, which may be misleading given the retrospective design and the strong potential for residual confounding, selection bias, and reverse causality. The conclusions should be tempered to clearly distinguish association from causation.

2. Methodological limitations and potential bias

- Late referral is defined retrospectively based on time to KRT initiation. This definition inherently excludes patients who die before reaching KRT, introducing survivor bias. Also, patients referred early may represent a systematically sicker group with greater comorbidity burden, which itself drives mortality risk, independent of referral timing. This limitation should be more explicitly acknowledged, particularly when interpreting survival differences after KRT.

- Patients with higher comorbidity burden, more rapid decline, or complex disease are more likely to be referred earlier. This may explain the observed higher mortality within and more than 12 months in the “early referral” group. Although comorbidity burden is discussed descriptively, it is not adequately controlled for in the final multivariable model.

- The study includes only patients already referred to public nephrology clinics in Queensland. Patients managed exclusively in primary care or private settings are not represented.

As such, the findings may not be generalizable to all CKD populations or to healthcare systems outside Australia.

3. Data Analysis and Interpretation

- Please show the table of univariate results and the final Cox model.

- It is unclear why other clinically important variables (e.g., diabetes, socioeconomic status, Indigenous status, comorbidity score as a composite) were excluded beyond a univariate p-value threshold. This approach may lead to model under-adjustment and overestimation of the apparent effect of referral timing.

- Maybe include an additional adjusted model incorporating comorbidity score and socioeconomic status, given their central importance to the study’s conclusions.

- Clarify whether proportional hazards assumptions were tested.

4. Interpretation and Conclusions

- The conclusion that referral timing “may no longer be reliable” as a determinant of outcomes is provocative but somewhat overstated given the observational nature of the data.

- The evidence more strongly supports the conclusion that comorbidity burden and socioeconomic disadvantage are dominant predictors of mortality, rather than that referral timing is unimportant.

Minor comments

1. Abstract: The Results section should more clearly state that the apparent survival advantage in late referral is likely confounded by comorbidity burden.

- Consider adding adjusted variables included in the final Cox model with p-value from table 2.

2. Terminology: Ensure consistent use of “early,” “timely,” and “late” referral throughout the manuscript.

- Clarify whether “early referral” always refers to >12 months before KRT.

3. Figures and Tables:

The figure showing the distribution of deaths by socioeconomic status should be revised to allow comparison between early and late referral groups.

Table 2 should present both univariate and multivariable analyses.

Table 3, which compares early versus late referral, would benefit from the inclusion of p-values for key baseline differences.

4. Missing clinical variables

Acknowledge the absence of data on dialysis modality, vascular access type, and cause of death as important unmeasured confounders.

5. References

Minor inconsistencies in formatting and typographical errors (e.g., spacing, hyphenation) should be corrected.

Language and Style

Extensive English editing is required to improve clarity, cohesion, and overall readability.

**********

what does this mean?). If published, this will include your full peer review and any attached files.

If you choose “no”, your identity will remain anonymous but your review may still be made public.

**Do you want your identity to be public for this peer review?**  For information about this choice, including consent withdrawal, please see our Privacy Policy

Reviewer #1: **Yes:** Wisit Kaewput

Reviewer #2: No

**********

[NOTE: If reviewer comments were submitted as an attachment file, they will be attached to this email and accessible via the submission site. Please log into your account, locate the manuscript record, and check for the action link "View Attachments". If this link does not appear, there are no attachment files.]

To ensure your figures meet our technical requirements, please review our figure guidelines: https://journals.plos.org/plosone/s/figures

You may also use PLOS’s free figure tool, NAAS, to help you prepare publication quality figures: https://journals.plos.org/plosone/s/figures#loc-tools-for-figure-preparation.

NAAS will assess whether your figures meet our technical requirements by comparing each figure against our figure specifications.

Attachment Submitted filename: Review_plosone_15122025.docx

# Author comment: Author response to Decision Letter 1

**DOI:** 10.1371/journal.pone.0338001.r002

16 Feb 2026

We thank the reviewers for the valuable feedback which has strengthened the manuscript. The document 'Response to Reviewers' that contains our responses has been uploaded with the rest of the documents supporting our manuscript.

Attachment Submitted filename: Response to Reviewers.docx

# Peer review: Decision Letter 1

**DOI:** 10.1371/journal.pone.0338001.r003

**Authors:** Jeerath Phannajit

14 May 2026

PLOS One

Dear Dr. Mutatiri,

Thank you for submitting your manuscript to PLOS ONE. After careful consideration, we feel that it has merit but does not fully meet PLOS ONE’s publication criteria as it currently stands. Therefore, we invite you to submit a revised version of the manuscript that addresses the points raised during the review process.

===================================

REQUIRED CHANGES (must be addressed for acceptance)

1. Analytical mitigation for survivor bias and selection bias: Both reviewers independently identify the lack of any analytical strategy to mitigate immortal time bias, survivor bias, and competing risks as the central methodological weakness. The definition of late referral based on time to KRT conditions on surviving to KRT, and the survival analysis anchored at KRT initiation further restricts the population to a selected subgroup. Acknowledgement of these limitations in the text, while appreciated, is insufficient on its own. We request that the authors attempt at least one complementary analysis to address this — for example, a competing-risks regression (Fine-Gray model) treating pre-KRT death as a competing event, or a landmark analysis. If the authors determine that such analyses are not feasible given the data structure or sample size, they must provide a clear methodological explanation of why this is the case and substantially strengthen the cautionary language in both the Abstract and the Discussion. At minimum, the Discussion should include an explicit statement that the absence of a statistically significant association cannot be interpreted as evidence that late referral is safe, and that the study is underpowered to exclude clinically meaningful harm. Authors should also acknowledge that restricting analysis to KRT starters may introduce collider bias if referral timing and comorbidity burden both determine who reaches KRT — this is not currently mentioned in the Limitations.

2. Clarify the Cox model population and time zero in the Methods: The Methods section currently states that the Cox model examined "associations between time of referral and the outcomes of death and KRT initiation" without specifying that the survival analysis was restricted to KRT starters (n=513) with time zero at KRT initiation. Please add an explicit sentence confirming this, for example: "Survival analysis was restricted to participants who commenced KRT (n=513), with time zero defined as the date of KRT initiation; deaths occurring prior to KRT were not included in this analysis."

3. Proportional hazards assumption: The manuscript does not state whether the proportional hazards assumption was tested. This is a standard requirement for Cox regression reporting. Please add a sentence in the Methods confirming whether the assumption was tested (e.g. using Schoenfeld residuals) and reporting the result.

4. Subgroup analyses at shorter referral thresholds: Reviewers note that the 12-month definition of late referral is broader than most prior studies, which typically use cut-offs of 3–6 months, and that this may dilute the observed association with adverse outcomes. We ask the authors to either (a) perform additional analyses using 3-month and/or 6-month thresholds to improve comparability with the existing literature, or (b) explicitly state in the Methods section why meaningful analysis at shorter thresholds is not feasible — for example, due to the very small number of participants who would qualify as "late referral" under stricter definitions, which would render any comparison statistically unreliable. Either response is acceptable provided it is clearly justified.

5. Correct the confidence interval error in Table 3: The Social Status row in Table 3 reports a 95% confidence interval of [1.75–1.58], in which the lower bound exceeds the upper bound. This is a transcription error and must be corrected.

6. Correct the Methods description of model-building: Table 2 correctly presents a full pre-specified model including all clinically important covariates. However, the Methods text still states that "the final model excluded variables with little univariate significance (p-value of >0.2)." This contradicts what is shown in Table 2. Please revise the Methods text to accurately describe the approach used — namely, that a pre-specified confounder set was retained in the model regardless of univariate significance.

7. The Conclusion states that the eGFR threshold "demonstrated similar prediction" to the KFRE. As noted by Reviewer 1, no formal measures of predictive performance (discrimination, calibration, or decision-curve analysis) were conducted, and this phrasing implies a comparative inferential claim that is not supported. Please revise to descriptive language, for example: "identified similar proportions of participants who progressed to KRT."

8. The rationale for the 12-month cut-off (elimination of vascular access as a confound) currently appears only in the Discussion. A brief statement of this justification should also be included in the Methods section at the point where late referral is defined.

9. Reference 18 contains "Octboer" (should be "October") and Reference 34 contains "sialysis access" (should be "dialysis access"). Please correct these and check the reference list for any remaining formatting inconsistencies.

RECOMMENDED CHANGES

10. Discussion framing — absence of evidence: Although the revised conclusion is more measured than the original, some language in the Discussion (for example, describing the non-significant Kaplan-Meier trend as warranting "clinical consideration") may still give readers the impression that the data support a clinically meaningful signal. We recommend adding a clear statement such as: "Absence of a statistically significant association should not be interpreted as evidence of absence of harm; this study was not adequately powered to exclude a clinically important mortality difference between referral groups."

11. Minor inconsistencies in the use of "referral date" versus "first nephrology visit" persist in the Abstract. Please review and ensure uniform terminology throughout.

12. Table 4: The UACR mean and SD for the early referral group are presented as "102.0 (145.7)" without the "SD" label, inconsistent with the column header and other rows. Please standardise.

===================================

Please submit your revised manuscript by Jun 28 2026 11:59PM. If you will need more time than this to complete your revisions, please reply to this message or contact the journal office at plosone@plos.org. When you're ready to submit your revision, log on to https://www.editorialmanager.com/pone/ and select the 'Submissions Needing Revision' folder to locate your manuscript file.

Please include the following items when submitting your revised manuscript:

- A letter that responds to each point raised by the academic editor and reviewer(s). You should upload this letter as a separate file labeled 'Response to Reviewers'.
- A marked-up copy of your manuscript that highlights changes made to the original version. You should upload this as a separate file labeled 'Revised Manuscript with Track Changes'.
- An unmarked version of your revised paper without tracked changes. You should upload this as a separate file labeled 'Manuscript'.

If you would like to make changes to your financial disclosure, please include your updated statement in your cover letter. Guidelines for resubmitting your figure files are available below the reviewer comments at the end of this letter.

If applicable, we recommend that you deposit your laboratory protocols in protocols.io to enhance the reproducibility of your results. Protocols.io assigns your protocol its own identifier (DOI) so that it can be cited independently in the future. For instructions see: https://journals.plos.org/plosone/s/submission-guidelines#loc-laboratory-protocols. Additionally, PLOS ONE offers an option for publishing peer-reviewed Lab Protocol articles, which describe protocols hosted on protocols.io. Read more information on sharing protocols at https://plos.org/protocols?utm_medium=editorial-email&utm_source=authorletters&utm_campaign=protocols.

As the corresponding author, your ORCID iD is verified in the submission system and will appear in the published article. PLOS supports the use of ORCID, and we encourage all coauthors to register for an ORCID iD and use it as well. Please encourage your coauthors to verify their ORCID iD within the submission system before final acceptance, as unverified ORCID iDs will not appear in the published article. *Only*  the individual author can complete the verification step; PLOS staff *cannot*  verify ORCID iDs on behalf of authors.

We look forward to receiving your revised manuscript.

Kind regards,

Jeerath Phannajit, M.D, Ph.D.

Academic Editor

PLOS One

Journal Requirements:

If the reviewer comments include a recommendation to cite specific previously published works, please review and evaluate these publications to determine whether they are relevant and should be cited. There is no requirement to cite these works unless the editor has indicated otherwise.

[Note: HTML markup is below. Please do not edit.]

Reviewers' comments:

Reviewer's Responses to Questions

**Comments to the Author**

Reviewer #1: (No Response)

Reviewer #2: All comments have been addressed

**********

2. Is the manuscript technically sound, and do the data support the conclusions?

Reviewer #1: Yes

Reviewer #2: Partly

**********

3. Has the statistical analysis been performed appropriately and rigorously?

Reviewer #1: Yes

Reviewer #2: No

**********

4. Have the authors made all data underlying the findings in their manuscript fully available?

The PLOS Data policy

Reviewer #1: Yes

Reviewer #2: Yes

**********

5. Is the manuscript presented in an intelligible fashion and written in standard English?

Reviewer #1: Yes

Reviewer #2: Yes

**********

Reviewer #1: This manuscript addresses an important and clinically relevant question regarding referral patterns in chronic kidney disease and their relationship with patient outcomes. The attempt to re-examine conventional time-based definitions of “late referral” and to move toward a more risk-based conceptual framework is thoughtful and aligns with evolving perspectives in CKD care. The authors have clearly made a genuine effort to respond to prior reviewer comments, particularly by clarifying terminology, moderating some interpretations, and expanding the discussion of limitations.

However, despite these improvements, several important methodological concerns remain insufficiently addressed, and these limit confidence in the validity and interpretation of the findings. Most notably, the definition of “late referral” continues to be derived retrospectively based on time to KRT initiation. This approach inherently conditions on future events and excludes patients who die prior to reaching KRT, thereby introducing selection and immortal time bias. Although the authors acknowledge these limitations, the current revision does not include any analytical strategy to mitigate them. As a result, the primary exposure remains difficult to interpret in a causal framework, and the observed associations may be substantially biased.

Related to this, the clarification that the survival analysis is anchored at the time of KRT initiation is helpful but raises additional concerns. Restricting the analysis to patients who survive to KRT introduces a selected population and may lead to collider bias, particularly if referral timing and comorbidity burden both influence progression to KRT. The manuscript would benefit from a more explicit justification of this design choice, along with consideration of complementary analyses that account for the pre-KRT period or competing risks such as death prior to KRT. At minimum, the implications of this conditioning should be more clearly articulated.

Concerns regarding confounding also remain. While the inclusion of a comorbidity score represents an improvement, the use of an unweighted Charlson index may not adequately capture differences in disease severity, and residual confounding is likely substantial. In addition, there remains some ambiguity regarding the model-building strategy, particularly whether variables were selected based on prior knowledge or data-driven approaches. Clarifying this point and ensuring that key clinical confounders are consistently included in the primary model would strengthen the analysis and improve interpretability.

The interpretation of the findings has been partially moderated, which is appreciated. Nevertheless, some statements still risk overemphasizing the apparent lack of harm, or even potential benefit, associated with later referral. Given the observational design and the multiple sources of bias discussed above, it is important that the conclusions consistently emphasize uncertainty and avoid suggesting that referral timing is unimportant. A more cautious framing that highlights the role of confounding, selection effects, and competing risks would be more appropriate.

The analysis of KFRE also remains limited. Although the terminology has been adjusted, the current presentation does not constitute a true evaluation of predictive performance. Without appropriate measures of discrimination or calibration, comparisons between KFRE and eGFR-based thresholds should either be removed or clearly described as descriptive rather than inferential.

Finally, several additional issues warrant further clarification. The relatively small number of late referral patients and outcome events raises concerns about statistical stability, and this should be more explicitly addressed, ideally with additional sensitivity analyses or stronger caution in interpretation. The handling of missing data, particularly for UACR, also remains suboptimal and may introduce bias; a clearer explanation of the analytical approach and its implications is needed. Issues of generalizability, given the restriction to public nephrology clinics, and the potential limitations of the comorbidity measure should be further elaborated in the discussion.

Reviewer #2: Thank you, Mutatiri et al., for the revised manuscript evaluating referral patterns among CKD.QLD Registry participants, with a focus on timing of referral and associated clinical outcomes. The topic is clinically relevant and timely, particularly in the context of evolving CKD management and increasing emphasis on risk-based care.

However, despite improvements, there remain important concerns regarding internal consistency of results, methodological limitations, and interpretation of findings, particularly the reported inverse association between late referral and mortality. These issues must be addressed before the manuscript can be considered for publication.

Suggestions for major revisions

Review Comments to the Author

Issues that need to be addressed include the following:

Major Comments

1. Overall significance and interpretation of the main finding

The finding that “late referral” is not associated with worse outcomes—and may even appear protective—is counterintuitive and challenges established evidence. The manuscript risks overstating this finding.

The observed association is likely influenced by residual confounding, selection bias, and reverse causality. This study also shows an imbalance between early referrals (448 cases) and late referrals (60 cases).

The definition of late referral used in this study (initiation of KRT within 12 months of the first nephrology visit) is broader than that in most prior studies, which commonly apply cut-offs of 3–6 months. While this approach may improve sensitivity in capturing delayed care, it could also dilute the association with adverse outcomes. Importantly, a large body of evidence has consistently demonstrated that late referral is associated with increased mortality, likely due to inadequate pre-dialysis preparation and higher rates of unplanned dialysis initiation.

Therefore, the authors should justify their chosen definition and consider additional subgroup analyses using shorter cut-offs to enhance comparability with existing literature such as in previous study, there was significantly increased overall mortality in the late referral group as compared with the early referral group (relative risk 1.99; 95% confidence interval [CI], 1.66 to 2.39, P <.0001).

https://pubmed.ncbi.nlm.nih.gov/18060927/

2. Methodological limitations and potential bias

Late referral is defined based on time to KRT, excluding patients who die before KRT and introducing survivor bias.

Patients referred earlier are likely sicker (higher comorbidity), creating confounding by indication.

Recommendation: Expand discussion of bias and clarify its impact on findings. Acknowledge limited generalisability.

3. Data analysis and internal consistency

There are inconsistencies in reported results, including hazard ratios and significance.

Subgroup analysis using cut-offs of 3 or 6 months may be needed.

https://pmc.ncbi.nlm.nih.gov/articles/PMC11737272/

Variable selection based on univariate p-values may lead to under-adjustment.

Recommendation: Clearly define models, include full tables, and ensure consistency across manuscript.

4. Interpretation of results and conclusions

The conclusion that referral timing may no longer be reliable is overstated.

Recommendation: Emphasise uncertainty and highlight comorbidity and socioeconomic factors as key predictors.

5. Definition of late referral

The 12-month definition is not sufficiently justified.

Alternative cut-offs of 3 or 6 months may be needed.

https://pmc.ncbi.nlm.nih.gov/articles/PMC11737272/

Recommendation: Justify threshold or include sensitivity analyses.

6. Role of KFRE and risk-based referral

KFRE analysis is underdeveloped and not integrated into main models.

Recommendation: Strengthen analysis or moderate conclusions.

Minor Comments

1. Abstract: Clarify confounding by comorbidity.

2. Terminology: Ensure consistency of referral definitions.

3. Tables/Figures: Improve formatting and include p-values.

4. Missing variables: Acknowledge absence of modality, access, cause of death.

5. Data quality: Correct typographical and formatting errors.

**********

what does this mean?). If published, this will include your full peer review and any attached files.

If you choose “no”, your identity will remain anonymous but your review may still be made public.

**Do you want your identity to be public for this peer review?**  For information about this choice, including consent withdrawal, please see our Privacy Policy

Reviewer #1: **Yes:** Wisit Kaewput, MD, FRCP(London), FASN, FAcadMEd

Reviewer #2: No

**********

[NOTE: If reviewer comments were submitted as an attachment file, they will be attached to this email and accessible via the submission site. Please log into your account, locate the manuscript record, and check for the action link "View Attachments". If this link does not appear, there are no attachment files.]

To ensure your figures meet our technical requirements, please review our figure guidelines: https://journals.plos.org/plosone/s/figures

You may also use PLOS’s free figure tool, NAAS, to help you prepare publication quality figures: https://journals.plos.org/plosone/s/figures#loc-tools-for-figure-preparation.

NAAS will assess whether your figures meet our technical requirements by comparing each figure against our figure specifications.

# Author comment: Author response to Decision Letter 2

**DOI:** 10.1371/journal.pone.0338001.r004

27 Jun 2026

Our responses to the Reviewers' comments have been uploaded with the rest of the documents.

Attachment Submitted filename: Response_to_Reviewers_auresp_2.docx

# Editor report: Decision Letter 2

**DOI:** 10.1371/journal.pone.0338001.r005

**Authors:** Jeerath Phannajit

6 Jul 2026

Dear Dr. Mutatiri,

Thank you for submitting your manuscript to PLOS ONE. After careful consideration, we feel that it has merit but does not fully meet PLOS ONE’s publication criteria as it currently stands. Therefore, we invite you to submit a revised version of the manuscript that addresses the points raised during the review process.

**Please revise the manuscript to address the following points:** plosone@plos.org. When you're ready to submit your revision, log on to https://www.editorialmanager.com/pone/ and select the 'Submissions Needing Revision' folder to locate your manuscript file.

- A letter that responds to each point raised by the academic editor and reviewer(s). You should upload this letter as a separate file labeled 'Response to Reviewers'.
- A marked-up copy of your manuscript that highlights changes made to the original version. You should upload this as a separate file labeled 'Revised Manuscript with Track Changes'.
- An unmarked version of your revised paper without tracked changes. You should upload this as a separate file labeled 'Manuscript'.

If you would like to make changes to your financial disclosure, please include your updated statement in your cover letter. Guidelines for resubmitting your figure files are available below the reviewer comments at the end of this letter.

If applicable, we recommend that you deposit your laboratory protocols in protocols.io to enhance the reproducibility of your results. Protocols.io assigns your protocol its own identifier (DOI) so that it can be cited independently in the future. For instructions see: https://journals.plos.org/plosone/s/submission-guidelines#loc-laboratory-protocols. Additionally, PLOS ONE offers an option for publishing peer-reviewed Lab Protocol articles, which describe protocols hosted on protocols.io. Read more information on sharing protocols at https://plos.org/protocols?utm_medium=editorial-email&utm_source=authorletters&utm_campaign=protocols.

As the corresponding author, your ORCID iD is verified in the submission system and will appear in the published article. PLOS supports the use of ORCID, and we encourage all coauthors to register for an ORCID iD and use it as well. Please encourage your coauthors to verify their ORCID iD within the submission system before final acceptance, as unverified ORCID iDs will not appear in the published article. *Only*  the individual author can complete the verification step; PLOS staff *cannot*  verify ORCID iDs on behalf of authors.

We look forward to receiving your revised manuscript.

Kind regards,

Jeerath Phannajit, M.D, Ph.D.

Academic Editor

PLOS One

Journal Requirements:

If the reviewer comments include a recommendation to cite specific previously published works, please review and evaluate these publications to determine whether they are relevant and should be cited. There is no requirement to cite these works unless the editor has indicated otherwise.

Please review your reference list to ensure that it is complete and correct. If you have cited papers that have been retracted, please include the rationale for doing so in the manuscript text, or remove these references and replace them with relevant current references. Any changes to the reference list should be mentioned in the rebuttal letter that accompanies your revised manuscript. If you need to cite a retracted article, indicate the article’s retracted status in the References list and also include a citation and full reference for the retraction notice.

[Note: HTML markup is below. Please do not edit.]

To ensure your figures meet our technical requirements, please review our figure guidelines: https://journals.plos.org/plosone/s/figures

You may also use PLOS’s free figure tool, NAAS, to help you prepare publication quality figures: https://journals.plos.org/plosone/s/figures#loc-tools-for-figure-preparation.

NAAS will assess whether your figures meet our technical requirements by comparing each figure against our figure specifications.

# Author comment: Author response to Decision Letter 3

**DOI:** 10.1371/journal.pone.0338001.r006

11 Jul 2026

The document with our responses to the Editor has been uploaded with the rest of the submission files.

Attachment Submitted filename: Response_to_Reviewers_auresp_3.docx

# Editor report: Decision Letter 3

**DOI:** 10.1371/journal.pone.0338001.r007

**Authors:** Jeerath Phannajit

29 Jul 2026

Referral patterns in the CKD.QLD Registry: a call for revisiting the definition of late referral

PONE-D-25-61252R3

Dear Dr. Mutatiri,

We’re pleased to inform you that your manuscript has been judged scientifically suitable for publication and will be formally accepted for publication once it meets all outstanding technical requirements.

Within one week, you’ll receive an e-mail detailing the required amendments. When these have been addressed, you’ll receive a formal acceptance letter and your manuscript will be scheduled for publication.

An invoice will be generated when your article is formally accepted. Please note, if your institution has a publishing partnership with PLOS and your article meets the relevant criteria, all or part of your publication costs will be covered. Please make sure your user information is up-to-date by logging into Editorial Manager at Editorial Manager® and clicking the ‘Update My Information' link at the top of the page. For questions related to billing, please contact billing support.

If your institution or institutions have a press office, please notify them about your upcoming paper to help maximize its impact. If they’ll be preparing press materials, please inform our press team as soon as possible -- no later than 48 hours after receiving the formal acceptance. Your manuscript will remain under strict press embargo until 2 pm Eastern Time on the date of publication. For more information, please contact onepress@plos.org.

Kind regards,

Jeerath Phannajit, M.D, Ph.D.

Academic Editor

PLOS One

Additional Editor Comments (optional):

Reviewers' comments:

# Editor report: Acceptance letter

**DOI:** 10.1371/journal.pone.0338001.r008

**Authors:** Jeerath Phannajit

PONE-D-25-61252R3

PLOS One

Dear Dr. Mutatiri,

I'm pleased to inform you that your manuscript has been deemed suitable for publication in PLOS One. Congratulations! Your manuscript is now being handed over to our production team.

At this stage, our production department will prepare your paper for publication. This includes ensuring the following:

* All references, tables, and figures are properly cited

* All relevant supporting information is included in the manuscript submission,

* There are no issues that prevent the paper from being properly typeset

You will receive further instructions from the production team, including instructions on how to review your proof when it is ready. Please keep in mind that we are working through a large volume of accepted articles, so please give us a few days to review your paper and let you know the next and final steps.

Lastly, if your institution or institutions have a press office, please let them know about your upcoming paper now to help maximize its impact. If they'll be preparing press materials, please inform our press team within the next 48 hours. Your manuscript will remain under strict press embargo until 2 pm Eastern Time on the date of publication. For more information, please contact onepress@plos.org.

You will receive an invoice from PLOS for your publication fee after your manuscript has reached the completed accept phase. If you receive an email requesting payment before acceptance or for any other service, this may be a phishing scheme. Learn how to identify phishing emails and protect your accounts at https://explore.plos.org/phishing.

If we can help with anything else, please email us at customercare@plos.org.

Thank you for submitting your work to PLOS One and supporting open access.

Kind regards,

PLOS ONE Editorial Office Staff

on behalf of

Dr. Jeerath Phannajit

Academic Editor

PLOS One
